# Lower Cretaceous fossils from China shed light on the ancestral body plan of crown softshell turtles (Trionychidae, Cryptodira)

**DOI:** 10.1038/s41598-017-04101-0

**Published:** 2017-07-27

**Authors:** Donald Brinkman, Márton Rabi, Lijun Zhao

**Affiliations:** 1 Royal Tyrrell Museum of Palaeontology, Box 7500, Drumheller, Alberta Canada T0J 0Y0; 2 Adjunct Professor, Department of Biological Sciences, University of Alberta, Edmonton, Alberta Canada; 3 Department of Earth Sciences, University of Torino, Via Valperga Caluso 35, 10125 Torino, Italy; 4 Institute of Geosciences, University of Tübingen, Sigwartstr. 10, 72076 Tübingen, Germany; 5 Zhejiang Museum of Natural History, No. 71 Jiaogong Road, Hangzhou, Zhejiang China; 6 Present Address: Zhejiang Museum of Natural History, 6 Westlake Culture Square, Hangzhou, Zhejiang Province China

## Abstract

Pan-trionychids or softshell turtles are a highly specialized and widespread extant group of aquatic taxa with an evolutionary history that goes back to the Early Cretaceous. The earliest pan-trionychids had already fully developed the “classic” softshell turtle morphology and it has been impossible to resolve whether they are stem members of the family or are within the crown. This has hindered our understanding of the evolution of the two basic body plans of crown-trionychids. Thus it remains unclear whether the more heavily ossified shell of the cyclanorbines or the highly reduced trionychine morphotype is the ancestral condition for softshell turtles. A new pan-trionychid from the Early Cretaceous of Zhejiang, China, *Perochelys hengshanensis* sp. nov., allows a revision of softshell-turtle phylogeny. Equal character weighting resulted in a topology that is fundamentally inconsistent with molecular divergence date estimates of deeply nested extant species. In contrast, implied weighting retrieved Lower Cretaceous *Perochelys* spp. and *Petrochelys kyrgyzensis* as stem trionychids, which is fully consistent with their basal stratigraphic occurrence and an Aptian-Santonian molecular age estimate for crown-trionychids. These results indicate that the primitive morphology for soft-shell turtles is a poorly ossified shell like that of crown-trionychines and that shell re-ossification in cyclanorbines (including re-acquisition of peripheral elements) is secondary.

## Introduction

Trionychids (softshell turtles) are aberrant turtles with a greatly reduced, flat shell, elongated neck, enlarged hyoid, streamlined skull, proboscis, and a highly aquatic, cryptic lifestyle. Their anatomy is greatly specialized relative to other turtles in the absence of a peripheral ring in the carapace, the replacement of all keratinous scales by leathery skin, and in the development of callosities on the rudimentary plastral elements^1–3^. Furthermore, the trionychid shell is sculptured by pits and ridges and the internal structure is uniquely reinforced by plywood-like collagen fibre bundles^4^. Altogether, these specializations make softshell turtles agile, gape-feeding ambush predators that are well adapted for remaining submerged for prolonged time period through increased cutaneous respiration^1, 5, 6^. Trionychids and carettochelyids (now represented by *Carettochelys insculpta*) form a monophyletic group, Trionychia^2, 7, 8^. Trionychians were initially thought to be closely related to kinosternoids on the basis of the pattern of cranial canals^9, 10^. However, more recent molecular studies of relationships have indicated that kinosternoids are more closely related to chelydroids while Pan-Trionychia had a long independent history, originating in the Middle to Late Jurassic^11–13^. Recent studies are converging on an extinct clade, Adocusia, as the stem-lineage of Trionychia^10, 14^. An early divergence of the clade is furthermore supported by the occurrence of stem-trionychians in the Jurassic of China^14, 15^. Pan-trionychids are dominant components of turtle faunas in Laurasia since the Late Cretaceous, but their origin and early evolution remains obscure. A key question pertains to the morphological changes associated with the divergence of the group into the two main extant subclades, Trionychinae and Cyclanorbinae. Trionychines are characterized by a reduced plastron and carapace whereas cyclanorbines typically possess a more ossified carapace and enlarged callosities in the plastron and even a partial ring of carapacial ossicles (peripheral elements) in one lineage (*Lissemys* spp^2^.). Which of these two morphotypes is ancestral for softshell turtles and whether the ossicles in *Lissemys* spp. are homologous to the peripheral elements of other turtles likewise remains to be clarified^16^. To resolve these questions, the early fossil record of trionychids is critical.

The earliest pan-trionychids are known from the Early Cretaceous of Asia. *Kappachelys okurai* Hirayama *et al*.^17^ from the Lower Cretaceous (?upper Neocomian) of Japan was initially thought to be a basal trionychid on the basis of the presence of typical trionychid sculpture and absence of sulci on the costals, but was recently shown to be a more basal pan-trionychian^18^. “*Trionyx*” *kyrgyzensis* Nessov^19^ from the Albian of Kyrgyzstan on the other hand is unquestionably a pan-trionychid (recently put in its own genus, *Petrochelys* ^20^). It is represented by disarticulated remains of several individuals that allow a reconstruction of the carapace and plastron. “*Trionyx*” *jixiensis* Li *et al*.^21^, from the Aptian-Albian of Northeastern China (Heilongjiang Province) is represented by a carapace. A third species, *Perochelys lamadongensis* Li *et al*.^22^ is represented by a complete skeleton from the Aptian of Liaoning, Northeastern China. *Perochelys lamadongensis* demonstrated that the long list of remarkable traits that Meylan^2^ identified as characteristic of trionychids had already been acquired by the Early Cretaceous and the majority of the group has remained largely unchanged for the last 120 million years. Because of this, plus the high degree of homoplasy, Li *et al*.^22^ were unable to resolve whether these Early Cretaceous pan-trionychids were stem member of the family or were within the crown.

In this paper, a pan-trionychid of Early Cretaceous age from the Hengshan Formation of Zhejiang Province, Southeastern China is described. This specimen, an articulated skeleton seen in ventral view, is described as *Perochelys hengshanensis* sp. nov. As in *Perochelys lamadongensis*, the derived features of the trionychid skeleton^2^ are fully developed. However, features that are primitive compared to those seen in crown-group trionychids can also be recognized. Thus, as well as adding to the distribution and diversity of pan-trionychids in the Early Cretaceous, this specimen provides an opportunity to revise the phylogeny of Early Cretaceous members of this family and place them relative to the crown group.

Abbreviations: AMNH, American Museum of Natural History; CRI, Peter C. H. Pritchard Collection, Oviedo, Florida, USA; FMNH, Field Museum of Natural History; NHMUK, Natural History Museum, London; USNM, Smithsonian National Museum of Natural History; ZMNH, Zhejiang Museum of Natural History.

## Methods

A revised dataset of Li *et al*.^22^ and Joyce *et al*.^23^, which is in turn based on the morphological matrices of Meylan^2^, Joyce *et al*.^24^ and Joyce and Lyson^25, 26^ and the molecular backbone constraint of Engstrom *et al*.^8^ was the basis of the phylogenetic parsimony analysis. In addition, four new characters were added and some scorings were changed based on new observations. Multistate characters that form a morphocline were treated as ordered (see Supplementary Information). Taxon sampling was increased by the addition of *Perochelys hengshanensis* sp. nov., *Petrochelys kyrgyzensis* (Nessov^19^), *Kuhnemys maortuensis* (Yeh^27^), *Gobiapalone orlovi* Danilov *et al*.^28^. *Aspideretoides foveatus* (Leidy^29^) was rescored based on more complete material. The topology of the molecular backbone constraint used in previous morphological analyses is relatively poorly resolved^20, 21, 25^. We here employ a composite tree of more recent and better supported phylogenies^30–32^. Only nodes of at least 99% bootstrap/posterior probability values were constrained; all others were turned into a polytomy (see Supplementary Information for constraint topology). All fossil ingroup taxa were designated as floaters. Instead of a hypothetical outgroup used in previous studies^2, 20, 21, 25^ we added *Adocus lineolatus* Cope^33^ and *Carettochelys insculpta* Ramsay^34^ to the matrix to avoid assuming the ancestral condition a priori.

Due to high levels of homoplasy, trionychid relationships have been difficult to reliably resolve using morphology alone^2^ and even the advancement of molecular phylogenies has not provided a framework that allows the placement of key early fossil taxa such as *Perochelys lamadongensis* ^22^ to be stabilized. Indeed, we identified 22 characters that evolved at least three times independently when optimized to the molecular phylogeny of extant Trionychidae (see Supplementary Information). In order to overcome this issue, the analysis was run using implied weighting as well (with K value ranging between 3 and 9 and including all characters)^35, 36^. The dataset consisting of 84 osteological characters and 33 taxa was analyzed in TNT using the tree bisection reconnection algorithm with thousand replicates and ten trees saved per replicate. Standard bootstrap absolute frequency values were calculated using 1,000 bootstrap replicates in TNT. Bootstrapping under implied weights was done with symetric resampling with 33 change probability.

## Results

Systematic Palaeontology

TESTUDINES Batsch^37^

CRYPTODIRA Cope^38^

TRIONYCHIA Hummel^7^

PAN-TRIONYCHIDAE Joyce *et al*.^39^

*Perochelys* Li *et al*.^22^

*Perochelys hengshanensis* sp. nov.

The electronic version of this article in Portable Document Format (PDF) will represent a published work according to the ICZN, and hence the new name contained in the electronic version are effectively published under that Code from the electronic edition alone. This published work and the nomenclatural acts it contains have been registered in ZooBank, the online registration system for the ICZN. The ZooBank LSIDs (Life Science Identifiers) can be resolved and the associated information viewed through any standard web browser by appending the LSID to the prefix http://zoobank.org/. The LSID for this publication is: urn:lsid:zoobank.org:pub:969D99E1-3513-4D74-9496-E36BE04E1EF6. The online version of this work is archived and available from the PubMed Central digital repository.

### Holotype

ZMNH M8750: articulated skeleton in ventral view, missing the anterior and right three quarters of the skull, lateral half of the right plastron, and a section through the anterior end of the shell including most of the anterior edge of the shell and the posterior portion of the neck.

### Locality and Horizon

**Figure 1:**
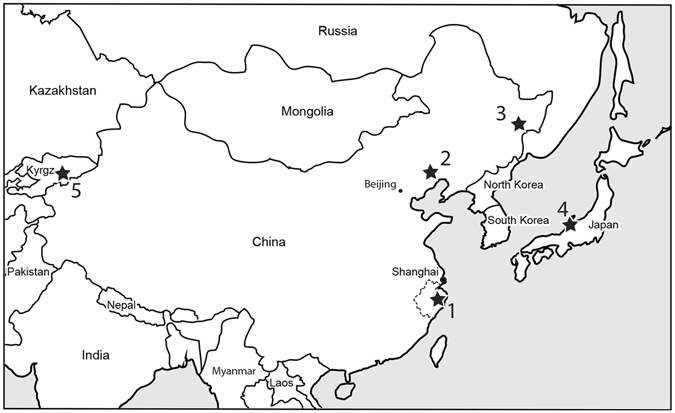
Map of Asia showing some of the Early Cretaceous trionychian localities. 1, *Perochelys hengshanensis*, ZMNH M8750, Hengshan Formation, Zhejiang Province, China. 2, *Perochelys lamadongensis*, Xiaotaize locality, Jiufotang Formation, Jianchang Country, Liaoning Province, China. 3, “*Trionyx*” *jixiensis*, Chengzihe Formation, Jixi Group, Hengshan District, Heilongjiang Province, China. 4, *Kappachelys okurai*, Akaiwa Formation? upper Neocomian, Japan. 5, *Petrochelys kyrgyzensis*, Kylodzhun locality, Alamyshik Fm., early middle Albian, Kyrgyzstan. Map data: Google, DigitalGlobe.

Hengshan Formation, Duntou Basin, Zhejiang Province, Peoples Republic of China (Fig. 1), at East longitude 119°52′16″ and North latitude 29°25′03″. The sedimentary age of the Hengshan Formation in the Duntou basin is 110.0 Ma−84.5 Ma^40^, and thus ranges from the Albian to the Santonian. The Hengshan Formation has been interpreted as deep, freshwater lake beds. The turtle specimen described here is from the Lower Cretaceous portion of the formation. Associated fauna includes fish, particularly *Neolepidotes* sp. which has also been collected in the lower part of the Hengshan Formation in the Duntou Basin.

### Etymology

The species is named after the Hengshan Formation, the formation from which the type and only specimen was collected.

### Diagnosis

Similar to *Perochelys lamadongensis* and *Petrochelys kyrgyzensis* in the proportions of the carapace and plastron, but differing in that the lateral end of the nuchal is broader, the mid-ventral plastral fenestra is shorter antero-posteriorly and wider medio-laterally, two lateral processes of the hyoplastron are separated by a groove that extends medially to the axillary notch, medial sutural edge of hyoplastron formed by numerous elongate, needle-like processes, the metischial process of the ischium is broader and points more strongly posteriorly, and the plastron lacks sculpturing.

## Description

### Skull

**Figure 2:**
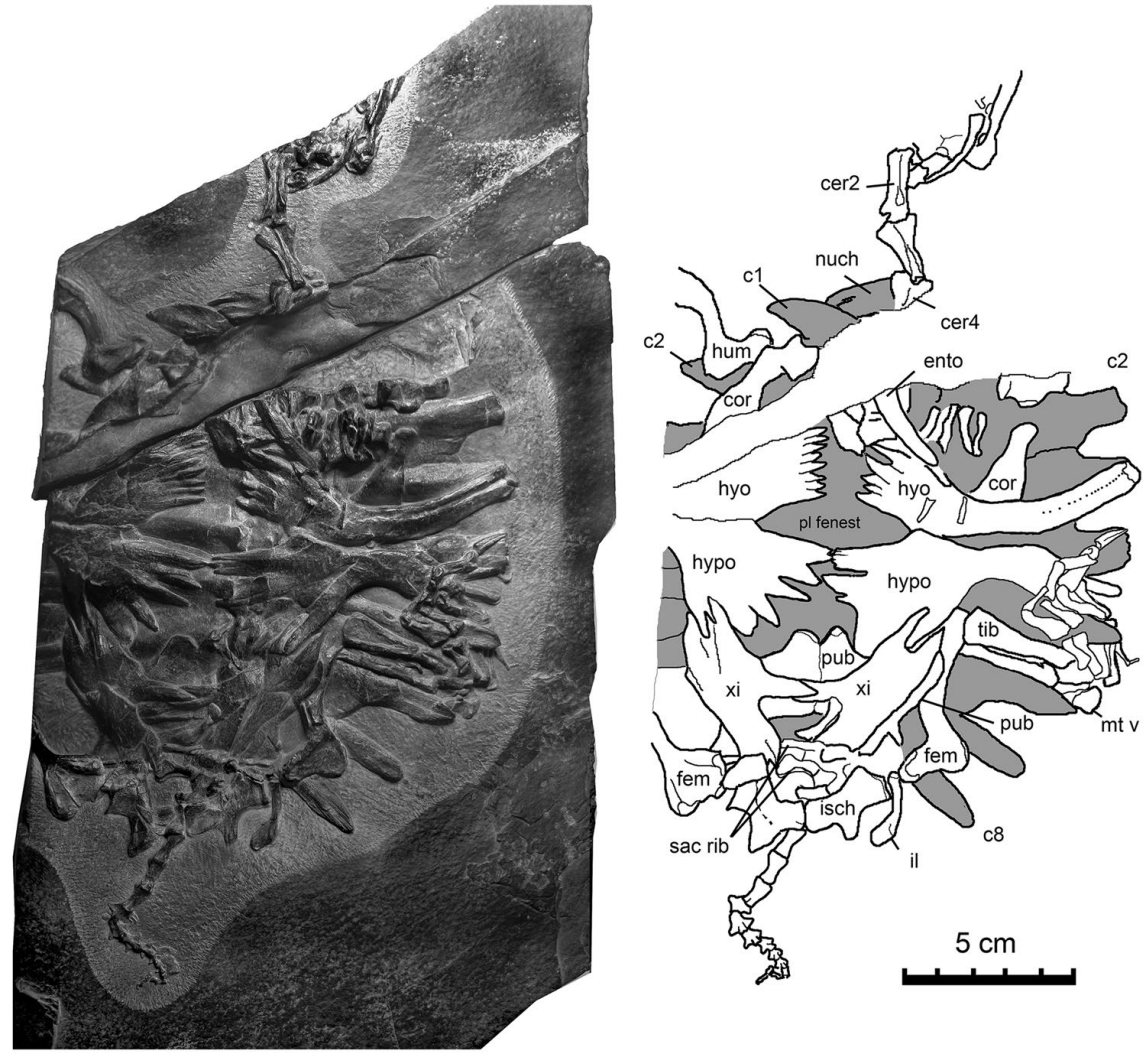
*Perochelys hengshanensis*, ZMNH M8750, Early Cretaceous, Hengshan Formation, Zhejiang Province, China. Photograph of specimen with line drawing. Exposed areas of carapace shaded. Abbreviations: c, costal; cer, cervical; cor, coracoid; ento, entoplastron; fem, femur; hum, humerus, hyo, hyoplastron, hypo, hypoplastron; il, ilium; Isch, ischium; mtv, fifth metatarsal; pl fenest, mid-ventral plastral fenestra; pub, pubis; sac rib, sacral rib; tib, tibia; xi, xiphiplastron.

**Figure 3:**
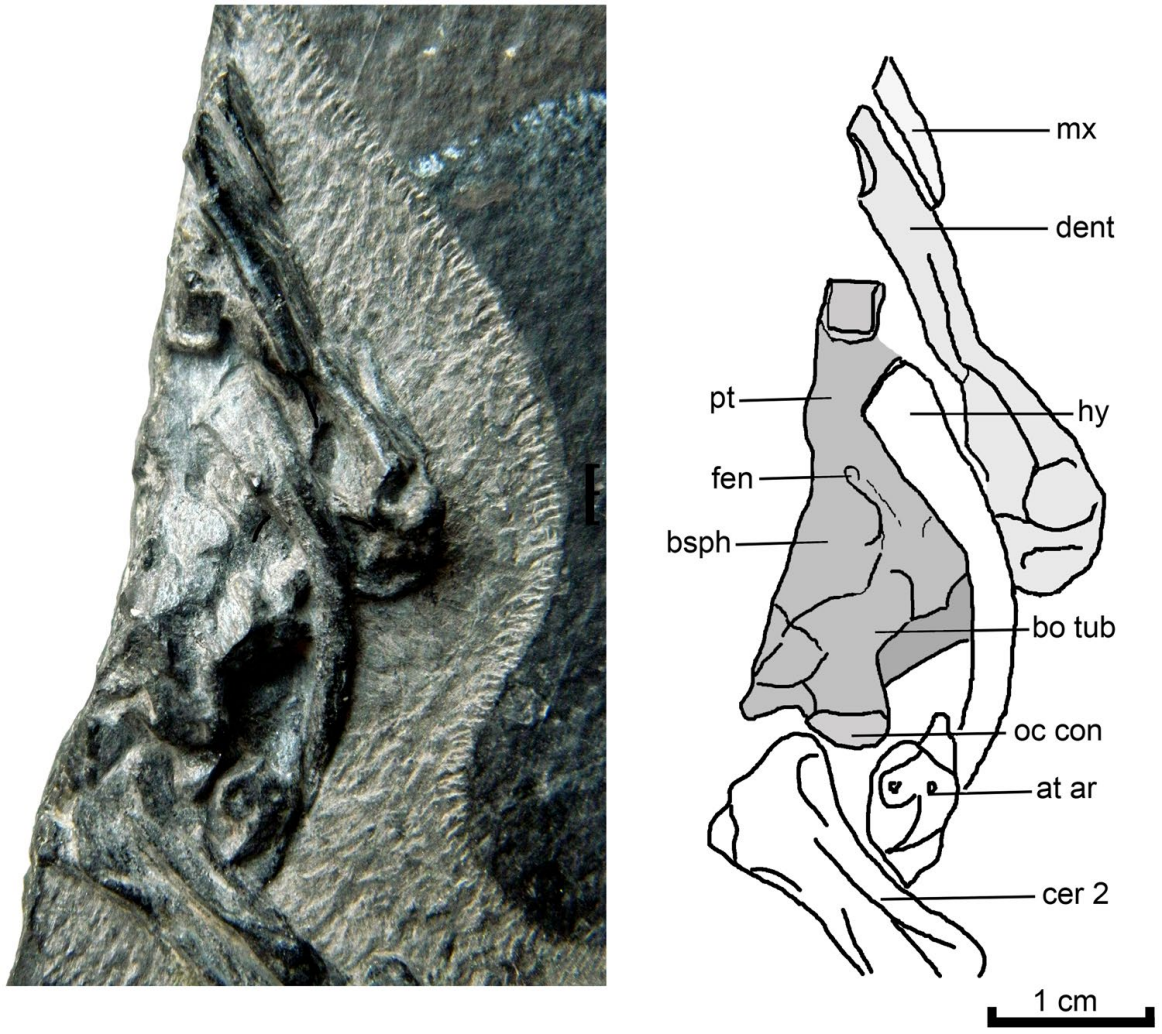
*Perochelys hengshanensis*, ZMNH M8750, Early Cretaceous, Hengshan Formation, Zhejiang Province, China. Close up of preserved portion of skull with line drawing. Abbreviations: at ar; atlas arch; bo tub, basioccipital tubercle;bsph, basisphenoid; fen, unknown fenestra or foramen; oc con, occipital condyle; pt, pterygoid.

The skull is represented by the back left quarter seen in ventral view (Figs 2 and 3). The lower jaw and hyoid are preserved in place and the basisphenoid and basioccipital are partially visible medial to the hyoid. A foramen is present between the basisphenoid and the pterygoid near the anterior end of the basisphenoid. The identity of this foramen is uncertain, it may be a foramen posterius canalis caroticus internus or it may be homologous to the opening in the palate of *Adocus* ^10^ that has been interpreted as a fenestra at the point where the carotid artery divides into the palatine branch (which extends forward between the pteryogid and basisphenoid) and the cerebral branch (which extends through the basisphenoid to enter the braincase)^41^. This fenestra has been interpreted as an intermediate stage in the transition between the primitive condition of having fully exposed carotid arteries ventral to the pterygoid and basisphenoid and a derived condition in which these are fully encased in canals within these bones^42–44^. Although the basioccipital is present, the number of hypoglossal foramina cannot be determined. The same fenestra has been termed the fenestra caroticus in turtles^44^.

### Neck

The first four cervical vertebrae are at least partially preserved (Fig. 2) and are very similar to that of extant trionychids. All are visible in ventral view. The atlas is represented by the arch (Fig. 3). As in other trionychids, the axis and anterior cervical vertebrae are elongate and weakly hourglass shaped, with the transverse process located at the anterior end of the centrum. The axis has a sharp mid-ventral ridge that extends along the anterior three quarters of the length of the centrum. Posterior to this, the ventral surface of the centrum is flattened. The third cervical has a strong mid-ventral keel along the anterior half of the centrum. Only the anterior portion of the fourth cervical vertebra is present.

### Sacrum

Two sacral vertebrae and their associated right sacral ribs are visible posterior to the right xiphiplastron (Fig. 2). The sacral vertebrae have a rounded ventral surface. The first sacral rib is relatively slender and rod-like, and the second is more robust and has an expanded lateral end.

### Tail

The tail is completely articulated (Fig. 2). Fifteen caudal centra are present. Fourteen of these are exposed posterior to the pelvis, and one is covered by the xiphiplastron. All of the exposed caudal vertebrae are seen in ventral view. The first three of these is nearly twice as long as wide. These are followed by centra that are subequal in length and width. The proportions of the posterior caudal centra match those of the fourth, but they decrease in size with the final three centra being tiny. The most anterior of the exposed centra has a rounded ventral surface and only weakly developed tubercles on its posterior edge. The tubercules become more strongly developed posteriorly and a pair of ridges extending to these tubercles becomes more distinct. However, the ridges do not extend the full length of the centrum until midway along the tail. Transverse processes are not visible on the three most anterior of the exposed centra, although this is likely because they are covered by matrix. The transverse process of the 5^th^ caudal vertebra (the fourth vertebra in the exposed portion of the tail) is located midway along the centrum. Its length is subequal or slightly shorter than the centrum. The following six vertebrae have similarly developed transverse processes. In its relative length, proportions of centra, and the development of transverse processes, the tail of *Perochelys hengshanensis* does not differ from younger trionychids.

### Pectoral girdle

The pectoral girdle is preserved in place. The right coracoid is preserved in articulation with the humerus and the base of the scapula. The left coracoid is visible except for the posterior end, which is covered by the hyoplastron. As is generally the case in trionychids, the coracoid has a constricted neck and an expanded shaft, giving the element a paddle-like shape.

### Pelvis

All three pelvic elements are at least partially visible. The pubis is largely covered by the xipiplastra, although its anterior end is visible between and anterior to the xiphiplastra and the lateral edge of the right pectineal process is visible just lateral to the xiphiplastra (Fig. 2). The exposed portions of the pubis demonstrate that, as in extant trionychids, the pectineal process was in the same plane as the more medial portion of the pubis and is larger than that surface.

The left ischium, which is fully exposed in ventral view, is a triradiate structure with robust medial and acetabular processes and a slender metischial process. The metischial processes are widely separated and point nearly directly posteriorly. The size and orientation of these processes differ from those in *Perochely lamadongensis*, which are longer, more strongly pointed, and slope postero-laterally. Among extant trionychids, the development of the metischial process varies^2^. Some, such as *Amyda cartilaginea* Boddaert^45^ have distinct metischial processes that are widely separated, while in others, such as *Apalone ferox* Schneider^46^, the metischian process is reduced to a slight posterior projection of the ischium.

The left ilium is visible lateral to the ischium (Fig. 2). This is a curved, rod-like element with an expanded dorsal end. A distinct bend is present between the shaft of the ilium and the dorsal expansion, and a tubercle is present on the antero-medial edge of the ilium at this bend. This contrasts with the typical condition in extant trionychids, where the ilium tapers smoothly from the shaft to the postero-dorsal tip. The expanded posterior end of the ilium in *Perochelys hengshanensis* is interpreted as a reduced iliac blade of more basal turtles. The tubercle may be homologous with the thelial process, which is present in more basal trionychians, such as *Adocus* sp.^10^.

### Fore limb

The right humerus is visible in postaxial view and is complete except for its distal end (Fig. 2). As in *Perochelys hengshanensis* and extant trionychids, it is strongly S-shaped with the upturned proximal end forming an angle of nearly 90° relative to the shaft. Elements of the left hand overlie the hyoplastron and entoplastron, although individual digits cannot be recognized.

### Hind limb

Both femora are at least partially preserved in articulation with the pelvis (Fig. 2). The right femur is represented by its proximal end, which is visible in ventral view. The left femur extends forward and is visible in postaxial view. It is complete, although the distal end is partly covered by the plastron and pubis. As in extant trionychids, the proximal end is upturned relative to the shaft, although not as strongly as the proximal end of the humerus. The trochanters, most clearly visible on the right femur, are subequal in size and are widely separated.

The left tibia and fibula, which are preserved in articulation with the femur and are visible in lateral view, are subequal to the femur in length.

The left foot is articulated and is preserved overlying the lateral end of the fifth costal. The metatarsals increase in length from the first to the fourth. The fifth has a short, “hooked” shape typical of turtles. The digital formula is 2,3,3,?,3. The number of phalanges in the fourth digit is uncertain because it is overlain by the third. The first three digits bear long claws.

### Carapace

The carapace is largely covered by the plastron and appendicular skeleton, but the free lateral ribs and portions of the lateral edge of the carapace are visible (Fig. 2). As in *Perochelys lamadongensis*, the carapace is small and round in outline.

The right portion of the nuchal is visible in ventral view, lateral to the third cervical vertebra. A deep groove is present near its anterior edge. Its lateral end lies against the anterior edge of the lateral ridge of the first costal. Compared with the corresponding lateral edge of the nuchal of *Perochelys lamadongensis*, the nuchal of *Perochelys hengshanensis* is longer antero-posteriorly and narrower medio-laterally.

The right first costal is represented by its lateral end. The free rib of the first costal is broad, has a straight posterior edge and a curved anterior edge. Striations extend to the lateral edge of the bone. The lateral free end of the rib of the second and more posterior costals are more symmetrical, the lateral tips of these ribs being located at about the middle of the costal. The free ends of the ribs of the seventh and eighth costals slope strongly posteriorly. Although the posterior edge of the eighth costal was not exposed, the slope of the posterior edge of the sixth costal indicates that it would have been small.

The neural series is covered by the plastron and girdles, so it is not known whether or not the neurals separated the eighth costal as in *Perochelys lamadongensis*.

### Plastron

**Figure 4:**
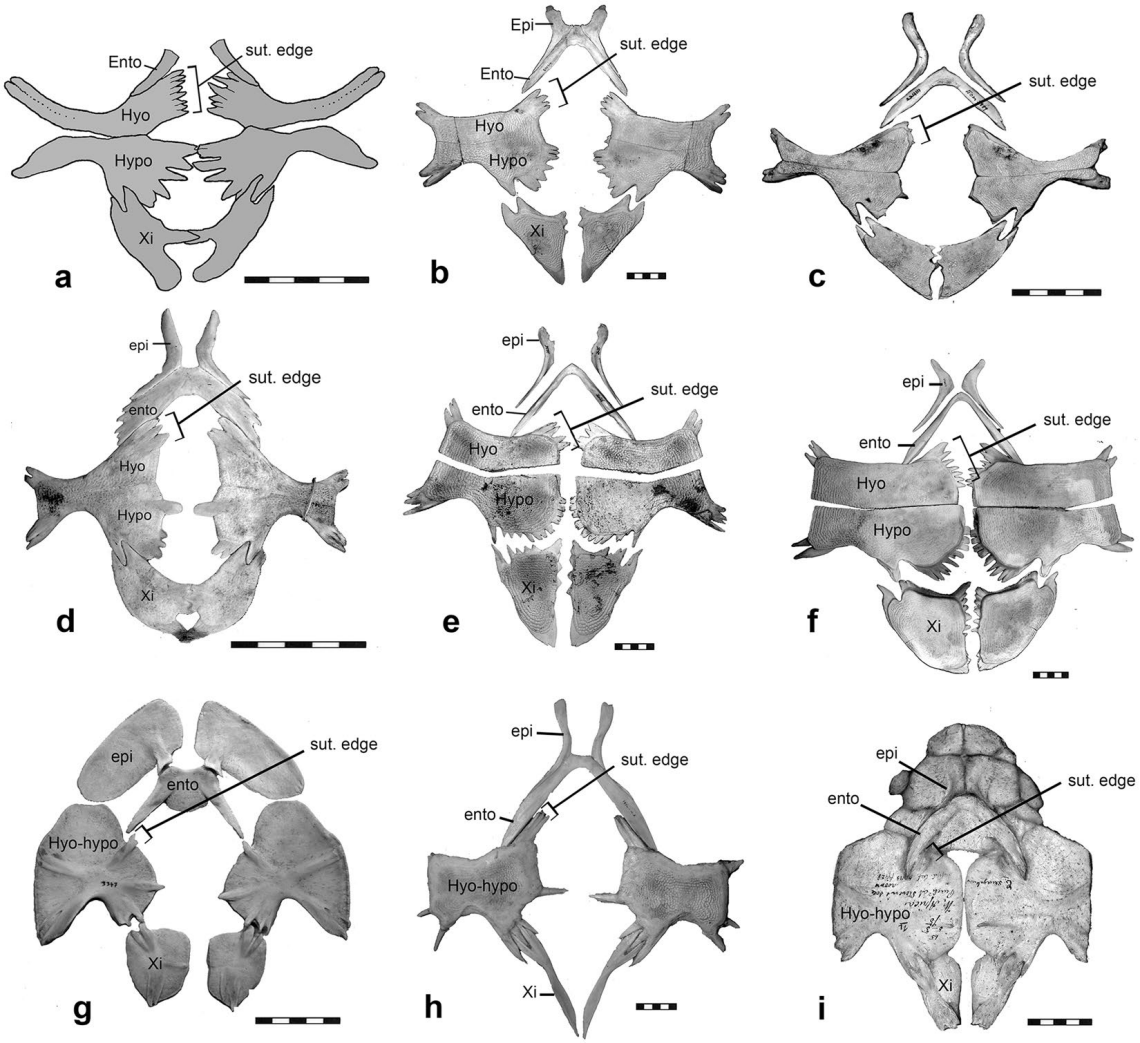
Plastron of *Perochelys hengshanensis*, compared with the plastron in extant trionychids. (**a**) line drawing of the plastron of *Perochelys hengshanensis*, ZMNH M8750, as preserved; (**b**) plastron of *Trionyx triunguis*, AMNH 80026; (**c**) plastron of juvenile individual of *Dogania subplana*, FMNH 224111; (**d**) plastron of *Pelodiscus sinensis* USNM 539335; (**e**) plastron of *Chitra indica* NHMUK 1926.12.16.1; (**f**) plastron of *Pelochelys briboni*, USNM 231523; (**g**) plastron of *Lissemys punctata*, CRI 2819; (**h**) plastron of subadult individual of *Cyclanorbis elegans*, NHMUK 1910, 6.3.1; (**i**) plastron of adult individual of *Cyclanorbis senegalensis*, NHMUK 65.5.3.75, seen in internal view. Scale bar equals 5 cm. Abbreviations: Epi, epiplastron; Ento, entoplastron; Hyo, hyoplastron; Hyo-hypo, fused hyo- and hypoplastron; Hypo, hypoplastron; Sut. edge, sutural edge of the hyoplastron; Xi, xiphipplastron.

The plastron is complete except for the epiplastron and part of the entoplastron (Figs 2 and 4). Distinct fenestrae are present medially between the hyo- and hypoplastra, the hypoplastra and xiphiplastra, and between the xiphiplastra. The medial fenestra between the hyo- and hypoplastra is oval in shape, narrow anteroposteriorly and wide medio-laterally, its length being half its width. This differs from that of *Perochelys lamadongensis* where the equivalent area is round in outline and is longer than wide. The fenestra between the xiphiplastra is posteriorly open. Laterally, the hyo- and hypoplastron are separated by a lateral plastral fenestra. This opening differs from the opening in *Perochelys lamadongensis* in being sub-triangular, rather than oval in shape and in extending further medially.

The ventral surface of the plastral elements lack the sculpturing typically present in extant trionychids. This differs from *Perochelys lamadongensis*, which has weakly developed sculpturing on the middle part of the hyoplastra, hypoplastra and xiphiplastra. *Petrochelys kyrgyzensis* from the Albian of Kyrgystan was initially described as lacking sculpturing on the plastron^19^, although it has subsequently been interpreted as having poorly developed sculpturing^22^. In the closely related *Petrochelys* cf. *kyrgizensis* from the Cenomanian of Uzbekistan both sculpted and smooth plastral elements are present^47^.

The epiplastron is missing. The posterior left end of the left entoplastron is preserved in articulation with the hyoplastron.

The hyo- and hypoplastron are reduced in size which makes the bridge very narrow, as in *Perochelys lamadongensis*. As in that taxon, the ratio between the length of the bridge and the length of the hypoplastron is about 30%. Among extant trionychids, these proportions are similar to those of *Dogania subplana* Geoffroy St.-Hilaire^48^ (Fig. 4c) and *Pelodiscus sinensis* Wiegmann^49^ (Fig. 4d).

The hyoplastron is a narrow element with an expanded medial portion. A distinct sutural edge formed by a series of long, slender, needle-like processes of subequal size is present on the medial edge of the bone anterior to the mid-plastral fenestra. Eight of these processes are present on the right hyoplastron. In extant trionychids, the homologous region is generally restricted to the anterior edge of the hyoplastron and the processes are much smaller (Fig. 4b–i). *Perochelys lamadongensis* is similar to *Perochelys hengshanensis* in having a sutural edge extending along the full length of the bone anterior to the mid-ventral fenestra but differs in that the processes are less needle-like.

The lateral end of the hyoplastron is not expanded relative to the bridge region. Two processes slightly separated at their tip are present on the antero-lateral edge of the element. A groove extends from the notch separating these two processes to the center of axillary notch. The extensive development of the groove was not observed in any other trionychid examined, and thus is interpreted as an autapomorphic feature of *Perochelys hengshanensis*.

The hypoplastron is similar to the hyoplastron in that the medial edge bears a series of spike-like processes that are subequal in size, although these are much more robust than the processes on the hyoplastron. These are most clearly seen on the right hypoplastron where they are evenly spaced and fan out from the center of the bone so the anterior process points medially and the posterior one points postero-medially. The anterior-most of these processes contact one another at the midline and the posterior-most process articulates with the xiphiplastron. The intermediate processes extend into the fenestra between the hypoplastron and the xiphiplastron. Although the medial edge of the hypoplastron of *Perochelys lamadongensis* is not well preserved, it appears to have a similar arrangement of processes. Among extant trionychids, the arrangement of the medial processes of the hypoplastron of *Perochelys hengshanensis* is similar to that in the extant genera *Pelochelys bibroni* (Fig. 4f), *Chitra indica* (Fig. 4e), and *Trionyx triunguis* (Fig. 4b) in that the processes are evenly spaced and do not strongly differ in size. In other trionychines the anterior-most process is much larger than the more posterior ones and separated from them by a larger gap (Fig. 4).

The xiphiplastron has three main branches, a lateral branch that articulates with the hypoplastron, an antero-medial branch that contacts the opposite xiphiplastron, and a posterior branch that borders the xiphiplastral fenestra. The posterior processes do not contact one another at the midline, so the fenestra is open posteriorly, although this may be because they have been slightly displaced. The strongly triradiate shape and extensive development of the fenestra between the xiphiplastra is a feature that is often present in subadult individuals of extant trionychids and lost in adults^50^.

## Phylogeny

**Figure 5:**
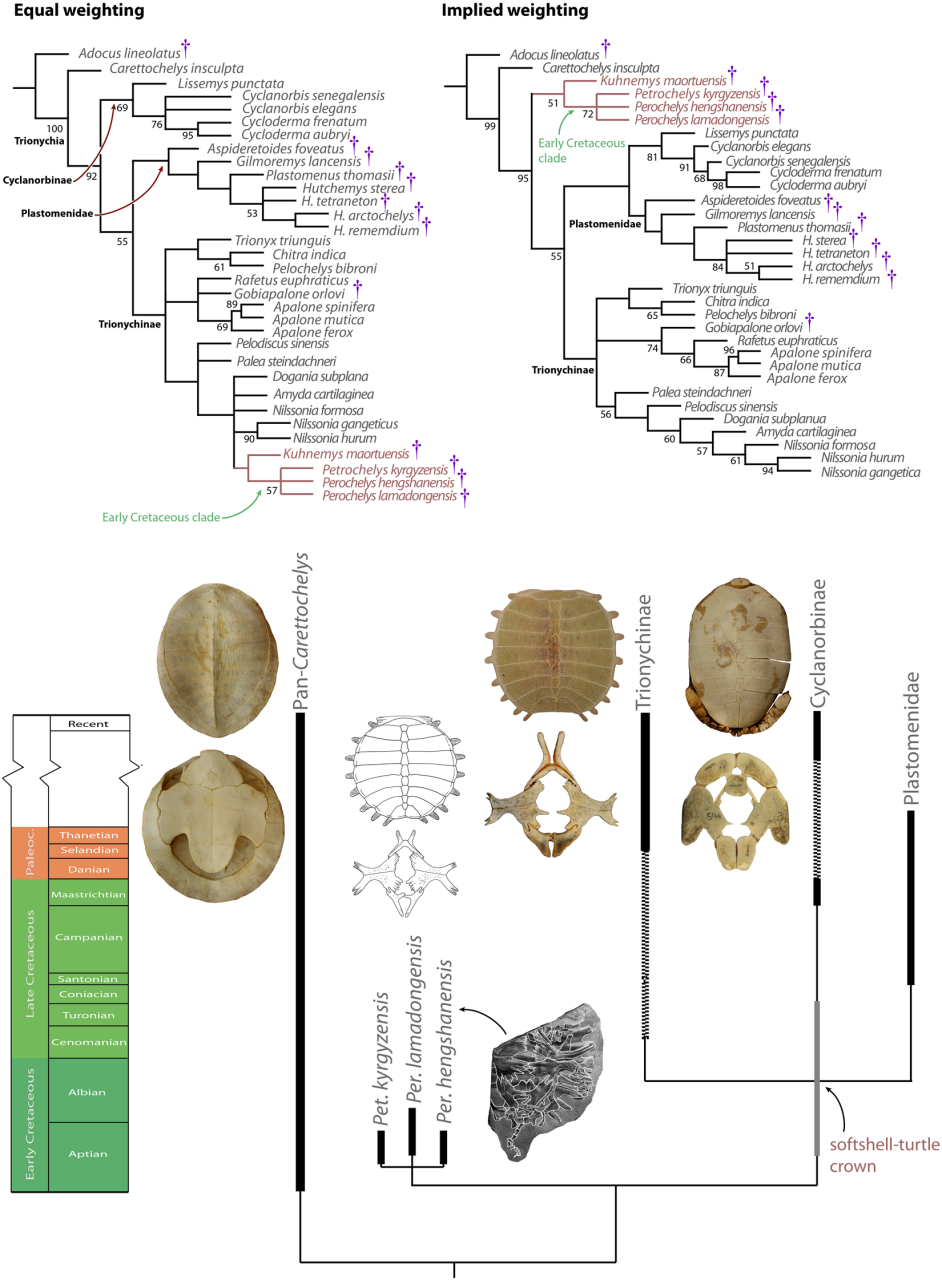
Top: Strict consensus trees of the parsimony analysis of the dataset using equal and implied weighting. Numbers denote standard bootstrap absolute frequency values above 50% (1000 replicates). Note that equal weighting results in a topology that is inconsistent with molecular divergence date estimates as well as the stratigraphic position of Early Cretaceous fossil taxa. Bottom: Inferred phylogeny and evolution of the total group of soft-shell turtles. Stem soft-shell turtle morphology indicates that the well-ossified morphotype was acquired secondarily in Cyclanorbinae. Stem softshell turtle morphology is illustrated by *Petrochelys kyrgyzensis* Nessov^19^.

The parsimony analysis with molecular scaffold and morphological characters sample recovered 24 trees of 321 steps (CI = 0.383; RI = 0.589) (Fig. 5). The Cretaceous *Petrochelys kyrgyzensis, Perochelys hengshanensis*, *P*. *lamadongensis*, and *Kuhnemys maortuensis* are placed in a highly controversial, deep position within Trionychinae either as a sister clade of *Nilssonia formosa* Gray^51^ or that of *Dogania* + *Amyda* + *Nilssonia* spp. (synapomorphies of the former clade: jugal-parietal contact on skull surface present, jugal contacts squamosal in one half of sample; of the latter clade: nine neurals; only costals 8 meeting at midline). However, implied weighting (K value = 3–5) recovers *Perochelys* spp., *Petrochelys kyrgyzensis*, and *Kuhnemys maortuensis* on the stem of crown group Trionychidae (6 trees; CI = 0.380, RI = 0.583). This position is supported by 1) the placement of dorsal vertebra 1 at the posterior edge of the nuchal; 2) expanded dorsal end of ilium, and 3) poorly ossified basibranchials. Further increase of K value places the Early Cretaceous taxa with *N. formosa*. See Supplementary Information for list of synapomorphies.

## Discussion

### Taxonomy

*Perochelys hengshanensis* is similar to *P. lamadongensis* and *Petrochelys kyrgyzensis* in the presence of a circular carapace and a plastron with narrow bridge and strongly triradiate xiphiplastron with a long, narrow antero-medial process that contacts its opposite at the midline.

*Perochelys hengshanensis* is distinct from *Perochelys lamadongensis* in six features. 1) The lateral end of the nuchal differs in being broad and having a groove on its ventral surface, rather than coming to a relatively sharp point as in *Perochelys hengshanensis*. 2) The size and orientation of the metischial processes differ in being broader and pointing more strongly posteriorly in *Perochelys hengshanensis*. 3) The development of sculpturing on the ventral surface of the plastron differs: in *Perochelys hengshanensis* sculpturing is absence, while in *Perochelys lamadongensis* it is present, although weakly developed. 4) The shape of the mid-ventral plastral fenestra differs in being a mediolaterally elongate oval in *Perochelys hengshanensis* and more circular in *Perochelys lamadongensis*. 5) The hyoplastron differs in the shape of the processes on the medial edge of the hyoplastron. These are more slender and needle-like in *Perochelys hengshanensis* than they are in *Perochelys lamadongensis*. 6) The presence of a groove on the hyoplastron extending from the base of the two lateral processes to the axial notch is a feature in which *Perochelys hengshanensis* differs from *Perochelys lamadongensis*, as well as all other trionychids that were observed. Since the parsimony analysis placed *Perochelys hengshanensis* in a monophyletic clade with *Petrochelys kyrgyzensis* and *Perochelys lamadongensis*, we conservatively place *Perochelys hengshanensis* in the genus *Perochelys* rather than erecting a new genus for it.

### Early evolution of Pan-Trionychidae

Despite their well-known morphology, the position of the earliest fossil pan-trionychids has proven to be extremely unstable: a position as stem-trionychids or within almost any crown-trionychid has been found equally likely in recent phylogenetic analyses^22^. A frustrating consequence is that such exquisite fossils as *Perochelys lamadongensis* have contributed little to our understanding of softshell turtle origins. In particular, a major issue concerning the early evolution of softshell turtles remains unsolved: does the well-ossified cyclanorbine morphotype or the poorly-ossified trionychine morphotype represent the ancestral condition for the crown group of softshell turtles? A generally poor early fossil record and a conservative but highly homoplastic morphological evolution are considered responsible for this uncertainty^2, 11, 22^.

We made numerous improvements to previous morphology matrices, including the addition of outgroup taxa, increased taxon and character sampling, the revision of scorings, and the employment of a better resolved molecular scaffold (see Methods). Parsimony analysis of this matrix resulted in a stable position of *Perochelys* spp. and *Petrochelys kyrgyzensis* but placed them deeply nested within Trionychinae (as sister to *Nilssonia formosa* or to *Dogania* + *Amyda* + *Nilssonia*) producing a major conflict with molecular divergence date estimates and the fossil record of crown-trionychines^3, 28, 30, 32, 51, 52^. The reason for this analytical artifact is apparently high levels of homoplasy and we identified 22 morphological characters that evolve at least three times independently when optimized on the molecular phylogeny of extant taxa. Although these characters have been in use since the pioneering systematic work of Meylan^2^ we find it reasonable to use implied weighting because: (1) the molecular phylogeny reveals that these may be not reliable for phylogenetic reconstruction, and (2) three of four characters that are responsible for the unreasonably deep nesting of the Early Cretaceous fossils under equal weight are among the 22 characters with highest homoplasy. Instead of omitting these homoplastic characters, a less radical approach is provided by implied weighting of the entire dataset^35, 36^. By doing so, *Perochelys* spp. and *Petrochelys kyrgyzensis* are retrieved as stem-trionychids which is in great agreement with their basal stratigraphic occurrence (Fig. 5) and the inferred Asian origin of Pan-Trionychidae^51, 52^.

The results of the analysis using implied weighting are consistent with recent studies that found the extinct Cretaceous-Paleogene North American clade Plastomeninae as stem-cyclanorbines^24^. However, we note that morphological support for this relationship is more limited than previously thought. For example, based on the epiplastron in an undescribed specimen of *Hutchemys tetanetron* Joyce and Lyson^25^, specimen TMP 93.94.1, and figures of *H. sterea* published by Hutchison^53^, particularly his figs 3–9, we interpret plastomenines as having a J-shaped, rather than I-shaped epiplastron. Furthermore, because the sutural contact between the hyoplastra of trionychines as interpreted above differs from that of plastomenines, we anticipate that the position of plastomenines as stem-cyclanorbines is an analytical artifact resulting in part of the absence of early cylanorbine fossils. Inclusion of the poorly known Maastrichtian *Nemegtemys conflata* within Cyclanorbinae (previously proposed on the basis of character diagnosis^28^) is supported by our phylogenetic analysis (Supplemenaty Information) and more material of this taxon could provide insights into the placement of plastomenines.

### Role of heterochrony in the origin of softshell turtles

It is striking that three of the Early Cretaceous stem-trionychid from Asia, *Perochelys hengshanensis*, *Perochelys lamadongensis* and *Petrochelys kyrgyzensis* are similar in the presence of features that are usually considered juvenile characteristics, including a reduced plastron with very large plastral fenestrae, strongly triradiate xiphiplastron, and small size. Although these features are typical of juveniles^50^, Nessov^19^ interpreted *Petrochelys kyrgyzensis* to be represented by adult individuals. The type specimens of *Petrochelys hengshanensis* and *Perochelys lamadongensis* are also likely adult individuals based on the free ends of costals 3 to 5 being shorter than the more medial portion of the costals (see Supplementary Information). The presence of certain juvenile features in adult stem-trionychids is consistent with the suggestion that paedomorphosis was a significant process during the origin of the Pan-Trionychidae^50^.

## Conclusions

The revised phylogeny presented here supports a stem-trionychid position for the Early Cretaceous *Perochelys* spp. and *Petrochelys kyrgyzensis* which is fully consistent with a “Mid-Cretaceous” (Aptian-Santonian) molecular age estimate for crown-trionychids^11^ as well as an Asian origin for Pan-Trionychidae.

The position of *Perochelys* spp. along the stem-lineage of Trionychidae indicates that the primitive morphology for soft-shell turtles is a poorly ossified shell that is generally identical with the crown-trionychine skeletal morphotype. The well-ossified cyclanorbine and plastomenine shell morphotype consisting of extensively developed callosities therefore should be considered a secondary acquisition. The lack of a peripheral ring in the carapace of stem-trionychids also implies that the peripheral ossicles of *Lissemys* spp. were acquired secondarily, in contrast to earlier hypotheses^2, 54^. Non-homology is furthermore supported by a lack of a one-to-one correspondence between peripheral ossicles and ribs, and the presence of more than one center of ossification per ossicle in *Lissemys* spp. (though a deeper homology is possible at a developmental/genetic level^16^).

The emerging picture is that soft-shell turtles attained a moderately broad distribution in Asia by the time they appear in the fossil record in the Early Cretaceous. Their absence outside Asia at this time^51, 52^ and molecular clock dating^11^ suggests that the divergence of the group was not much earlier than their first appearance in the fossil record. The development of the highly specialized softshell skeletal anatomy therefore must have evolved very rapidly and remained largely unchanged ever since, except for a secondary re-ossification in most cyclanorbines. Juvenile characteristics in *Perochelys* spp. and *Petrochelys kyrgyzensis* suggest that heterochrony played role in the development of the softshell turtle body plan which is in turn consistent with their overall poorly ossified shell. Following their initial radiation, soft-shell turtles became more widespread and dominant in turtle faunas by the “mid-Cretaceous” and dispersed to North America in the Cenomanian^55^.

## Electronic supplementary material

Supplementary Information
