# Supplementary material for: Lower Cretaceous fossils from China shed light on the ancestral body plan of crown softshell turtles (Trionychidae, Cryptodira)
Source: Sci Rep. 2017 Jul 27;7:6719. doi: 10.1038/s41598-017-04101-0 (PMC5532300; doi:10.1038/s41598-017-04101-0)
Supplement: Supplementary file 1 — Supplementary Information [file 41598_2017_4101_MOESM1_ESM.pdf]

Supplementary Information

Lower Cretaceous fossils from China shed light on the ancestral body plan of crown softshell-turtles (Trionychidae, Cryptodira)

Donald B. Brinkman[1*], Márton Rabi[2*] & Zhao Lijun[3]

[1]1Royal Tyrrell Museum of Palaeontology, Box 7500, Drumheller, Alberta, Canada T0J 0Y0
[1]Adjunct Professor, Department of Biological Sciences, University of Alberta, Edmonton, Alberta
Email: don.brinkman@gov.ab.ca

[2]Department of Earth Sciences , University of Torino, Via Valperga Caluso 35, 10125 Torino, Italy
[2]Institute of Geosciences , University of Tübingen, Sigwartstr. 10, 72076 Tübingen, Germany
Email: iszkenderun@gmail.com

[3]Zhejiang Museum of Natural History , No.71, Jiaogong Road, Hangzhou, Zhejiang, China
Email: zhaolj@zmnh.com

Correspondence and requests for materials should be addressed to D.B.B (e-mail: don.brinkman@gov.ab.ca) or M. R. (e-mail: iszkenderun@gmail.com).

Table of Contents:

Supplementary Materials ………..………………………………………………………… 3

Institutional abbreviations ……….………………………………………………………… 4

Supplementary Data ……….……………………………………………………………….. 4

List of characters ………………………………………………………………………….. 4

List of homoplastic characters ……………………………………………………….. 14

Modifications to the matrix of Li et al. (2015) and Joyce et al. (2016) …….……. 15

Changes in the coding of *Aspideretoides foveatus* …………………………………… 18

Character matrix …………..…………………………………………………………. 20

Molecular backbone constraint ……………………………………………………….... 25

Ontogenetic ossification patterns of post- hatchling trionychids shells …...…………… 25

Phylogenetic position of *Nemegtemys conflata* ……………………...……………………. 28

Common synapomorphies under implied weighting (K value = 3) 29

Synapomorphies common to 8 trees, equal weighting 42

Supplementary References …………………………………...……………………… 56

Supplementary Materials

List of taxa used in the phylogenetic analysis with the source of data (either specimens or publications) that is the basis for the coding of the five new characters added to the data matrix.

Cycloderma aubryi: MNHM1930-362.
Pelochelys bibroni: USNM 231523; IVPP 537.
Amyda cartilaginea: USNM 22522; Siebenrock (1898).
Cyclanorbis elegans: NMW p272.
Rafetus euphraticus: NMW 93.10.14.1.; Meylan (1987).
Apalone ferox: USNM 71069.
Cycloderma frenatum: AMNH 110180.
Nilssonia gangeticus: USNM 293693, 267017; Meylan (1987).
Chitra indica: FMNH 224228; Meylan (1987).
Apalone mutica: UMMZ 155231.
Lissemys punctata: AMNH 108907; CRI 2819; Meylan (1987).
Cyclanorbis senegalensis: NHMUK 65.5.3.75.; Siebenrock (1898).
Pelodiscus sinensis: USNM 539335; NHMW 1865; Siebenrock (1898).
Apalone spinifera: YPM R 10892; Sheil (2003).
Dogania subplana: USNM 222523.
Trionyx triunguis: AMNH 36599.
Gilmoremys lancensis: Joyce and Lyson (2011) Fig. 9 – internal view.
Palea steindachneri: no access; scores unknown.
Nilssonia hurum: NHMUK 86.8.26.2; Meylan (1987).
Nilssonia formosus: no access; scores unknown.
Plastomenus thomasii: Hutchison (2009) Fig. 1.
Huchemys rememdium: Joyce et al. (2009) Figs. 2-4.
Huchemys arctochelys: Joyce et al. (2009) Figs. 5-9.
Huchemys tetraneton: Hutchison (2009) Figs. 21-26.
Huchemys sterea: Hutchison (2009) Figs. 3-9.
Perochelys lamadongensis: IVPP 18048; Li et al. (2015).
Perochelys hengshanensis: ZMNH M8750.
Petrochelys kyrgyzensis: Nessov (1995).
Carettochelys insculpta: NHMUK 1903.7.10.1; CRI 14; UF 49415.
Adocus lineolatus: Meylan and Gaffney (1989).
Nemegtemys conflata: Danilov et al. (2014).
Gobiapalone orlovi: Danilov et al. (2014).
Aspideretoides foveatus: TMP 81.24.7, complete skeleton; TMP 2005.12.557, skull; TMP 2000.12.2 partial skull.
Kuhnemys maortuensis: IVPP V 2864; Yeh (1965).

Institutional abbreviations

AMNH, American Museum of Natural History; New York, USA
CRI, Peter C. H. Pritchard Collection; Oviedo, Florida, USA
FMNH, Field Museum of Natural History; Chicago, Illinois, USA
IVPP, Institute of Vertebrate Paleontology and Palaeoanthropology, Beijing, China
MNHM, Muséum National d'Histoire Naturelle; Paris, France
NHMUK, Natural History Museum, London; United Kingdom
NMW, Naturhistorisches Museum Wien; Vienna, Austria
UF, Florida Museum of Natural History; Gainesville, USA
UMMZ, University of Michigan Museum of Zoology
USNM, Smithsonian National Museum of Natural History; Washington DC, USA
TMP, Royal Tyrrell Museum of Palaeontology, Drumheller, Alberta, Canada
YPM, Yale Peabody Museum; New Haven, Connecticut, USA
ZMNH, Zhejiang Museum of Natural History, Hangzhou, China

Supplementary Data

List of characters included in phylogenetic analysis. The character list is modified from the matrix of Li et al. (2015) and Joyce et al. (2016) which in turn were based on Meylan (1987), Joyce et al. (2009), and Joyce and Lyson (2011). In addition, 4 new characters are introduced here. The taxon-character matrix is uploaded as online supplementary information.

1) Width/length of nuchal bone (Meylan 1987: 1); ORDERED
1. less than 2
2. greater than 2
3. greater than 3
4. greater than 4

2) Anterior and posterior costiform processes of nuchal bone united (Meylan 1987: 2);
1. no
2. yes

3) Position of anterior edge of first body vertebra relative to nuchal bone (Meylan 1987: 3); ORDERED

1. posterior edge of nuchal
2. middle of nuchal
3. anterior edge of nuchal

4) First and second neurals fused (Meylan 1987: 4);

1. no
2. yes

5) Total number of peripherals (Meylan 1987: 5); ORDERED

1. 22
2. 20
3. 14-18
4. 0

6) Prenuchal bone (Meylan 1987: 7);

1. absent
2. present

7) Size of eighth pleurals (Meylan 1987: 8);

1. large
2. reduced or absent

8) Number of plastral callosities (Meylan 1987: 9);

1. seven
2. five
3. four
4. two
5. none

0. nine

9) Hyoplastra and hypoplastra fuse just after hatching (Meylan 1987: 10);

1. no
2. yes

10) Fusion of xiphiplastra (Meylan 1987: 12);

1. absent
2. present

11) Hypo-xiphiplastral union (Meylan 1987: 13);

1. xiphiplastra lateral to hypoplastra
2. hypoplastra lateral to xiphiplastra

12) Number of neurals (fused 1 and 2 counted as 2; Meylan 1987: 14); ORDERED

1. nine
2. eight or nine
3. eight
4. seven or eight
5. seven or fewer

13) Variability in position of neural reversal (Meylan 1987: 15);

1. always at same neural
2. always at adjacent neurals
3. highly variable

14) Pleurals (=costals) which meet at midline (Meylan 1987: 16);

1. eighth only
2. seventh and eighth or eighth only
3. sixth, seventh, and eighth or seventh and eighth
4. more than sixth, seventh, and eighth

0. none

15) Point of reversal of orientation of neurals (Meylan 1987: 17);

1. at neural eight
2. at neural seven
3. at neural six or seven
4. at neural six
5. at neural four, five, or six
6. (New state) no reversal

16) Suprascapular fontanelles (Meylan 1987: 18); ORDERED

1. closed at hatching
2. closed in large adults only
3. open throughout life

17) Epiplastron shape (Meylan 1987: 19);

1. J-shaped
2. I-shaped

18) Length epiplastra anterior to entoplastron contact (Meylan 1987: 20); ORDERED

1. short
2. intermediate

3. long

19) Depressions on eighth pleurals for contact of ilia (Meylan 1987: 21);
1. present
2. absent

20) Bridge length (Meylan 1987: 23);
1. long
2. short

21) Largest adult size 200 mm or less (disc length; Meylan 1987: 24);
1. no
2. yes

22) Carapace margin straight to concave posterolaterally (Meylan 1987: 25);
1. no
2. yes

23) Sexual dimorphism in disc length (Meylan 1987: 29);
1. no
2. yes

24) Jugal contacts squamosal (Meylan 1987: 32);
1. no
2. in one-half of sample

25) Jugal contacts parietal on skull surface (Meylan 1987: 34);
1. no
2. in one-half of sample
3. yes

26) Vomer contacts prefrontal (Meylan 1987: 36);
1. yes
2. no

27) Dorsal edge of aperture narium externum laterally emarginated (Meylan 1987: 41);
ORDERED
1. no
2. weakly
3. strongly

28) Dorsal edge of aperture narium externum medially emarginated (Meylan 1987: 42);
1. no

2. yes

29) Basisphenoid contacts palatines (Meylan 1987: 46);
1. no
2. yes

30) Vomer divides maxillae (Meylan 1987: 48);
1. yes
2. no

31) Vomer reaches intermaxillary foramen (Meylan 1987: 49);
1. yes
2. no

32) Vomer contacts basisphenoid (Meylan 1987: 51);
1. no
2. occasionally

33) Size of foramen palatinum posterius (Meylan 1987: 53);
1. large
2. small
3. small and divided
4. many small openings

34) Foramen palatinum posterius forms in (Meylan 1987: 54);
1. palatine and pterygoid and/or maxilla
2. palatine only

35) Foramen jugulare posterius (Meylan 1987: 58, modified by Joyce et al. 2016);
1. open
2. enclosed

36) Enclosed foramen jugulare posterius (Meylan 1987: 59, modified by Joyce et al. 2016);
1. enclosed by pterygoid
2. enclosed by opisthotic

37) Foramen posterius canalis carotici interni relative to lateral crest of basioccipital tubercle (Meylan 1987: 60); ORDERED
1. above
2. in it
3. below

38) Maxilla contacts frontal in front of orbit (Meylan 1987: 62);
1. no
2. yes

39) Exoccipital contacts pterygoid (Meylan 1987: 63);
1. no
2. yes

40) Basisphenoid shape (Meylan 1987: 64);
1. not medially constricted
2. occasionally medially constricted
3. medially constricted

41) Premaxilla absent (Meylan 1987: 65);
1. no
2. occasionally
3. usually

42) Vomer lost (Meylan 1987: 66);
1. no
2. yes

43) Jugal contacts orbit (Meylan 1987: 67);
1. yes
2. no

44) Epipterygoid, if present, contacts the palatine (Meylan 1987: 68);
1. yes
2. in ca. 50%
3. no

45) Contact between pterygoid and foramen nervi trigemini occurs when epipterygoid is present (Meylan 1987: 69);
1. yes
2. no

46) When epipterygoid is present pterygoid contacts foramen nervi trigemini (Meylan 1987: 70);
0. between epipterygoid and quadrate or not at all
1. between prootic and epipterygoid or not at all
2. between epipterygoid and parietal or not at all

47) Epipterygoid contacts prootic anterior to foramen nervi trigemini (Meylan 1987: 71);

1. no
2. in ca. 50%
3. yes

48) Epipterygoid contacts prootic posterior to foramen nervi trigemini (Meylan 1987: 72);
1. no
2. yes

49) Epipterygoid fuses to pterygoid (Meylan 1987: 73) ; ORDERED
1. in subadults
2. in adults only
3. never

50) Average ratio of intermaxillary foramen length to length primary palate (Meylan 1987: 74); ORDERED
0. 0.07
1. about 0.20 to 0.40
2. about 0.60

51) Postorbital bar relative to orbit (Meylan 1987: 75); ORDERED
0. about 2 times orbit diameter
1. about equal to orbit to 1/3 of orbit
2. less than 1% of orbit

52) Quadratojugal participates in processus trochlearis oticum (Meylan 1987: 76);
1. no
2. yes

53) Proportion of processus trochlearis oticum made up by parietal (Meylan 1987: 78);
1. 15.6% or less
2. 22.1% or more

54) Ventral keel on 8th cervical present and limited to posterior end (Meylan 1987: 87);
1. no
2. yes

55) Strong dorsal processes on cervicals (Meylan 1987: 88);
1. no
2. yes

56) Number of ossifications in corpus hyoidis (Meylan 1987: 90);

1. 1
2. 6
3. 8

57) Number of ossifications in comu branchiale II (Meylan 1987: 91);
1. 1 only
2. 2-6
3. 7 or more

58) Ossifications of comu branchiale II broad and strongly sutured (Meylan 1987: 92);
1. no
2. yes

59) Basihyals in close contact and projecting anteriorly (Meylan 1987: 93);
1. no
2. yes

60) Symphyseal ridge strong and present in a depression (Meylan 1987: 95);
1. no
2. yes

61) Foramen intermandibularis caudalis enclosed by prearticular (Meylan 1987: 98);
1. sometimes
2. never

62) Ilia curve medially (Meylan 1987: 100);
1. no
2. yes

63) Ischia extend into thyroid fenestra (Meylan 1987: 107);
1. yes
2. no

64) Metischial processes present and distinct (Meylan 1987: 109);
1. yes
2. no

65) Angle of acromion process to scapula approaches that of coracoid to acromion (Meylan 1987: 112);
1. no
2. yes

66) Coracoid longest of three pectoral processes (Meylan 1987: 113);

1. no
2. yes

67) Development of surface sculpturing of carapace and plastron (Joyce et al. 2009: 67);

0. all metaplastic portions of carapace and plastron have trionychid sculpturing;
1. trionychid pattern grades towards the center of carapacial and plastral disk to a smooth pattern, as developed in *Hutchemys rememdium* and *Hutchemys arctochelys*.
2. (New state). plastron sculpture greatly subdued or absent.

68) Nuchal notch (Joyce et al. 2009: 68);

0. anterior rim of nuchal convex or slightly notched;
1. anterior rim of carapace with deep nuchal notch, as developed in *H. rememdium*.

69) Shape of neural 1 and 2 (= neural 2 and 3 of Meylan 1987; Joyce et al. 2009: 69);

0. neurals 1 and 2 hexagonal with short posterior sides;
1. neural 1 circular to rectangular and neural 2 octagonal, as developed in *H. rememdium* and *H. arctochelys*.

70) Splitting of costals along distal margin (Joyce et al. 2009: 70);

0. costal rim rounded or graded;
1. dorsal rims split into separately protruding dorsal and visceral portions, as developed in *H. rememdium* and *H. arctochelys.*

71) Lateral notch in carapace at the level of costal 5 (Joyce et al. 2009: 71);

0. absent, lateral carapacial margin rounded;
1. present, lateral carapacial margin shows a waist, as developed in *H. arctochelys*.

72) Skin callosity developed on the visceral side of costals 6 and 7 (Joyce et al. 2009: 72);

0. absent, visceral side smooth;
1.present, visceral side sometimes develops a callosity, as seen in *H. arctochelys*.

73) Mid-line contact of hyoplastra, hypoplastra and xiphiplastra (Joyce et al. 2009: 73);

0. hyo-, hypo- and xiphiplastra do not contact another fully, even in adults;
1. hyo-, hypo and xiphiplastra contact another fully along the entire mid-line in adults, as developed in *H. rememdium* and *H. arctochelys.*

74) Shape of deep portion of entoplastron (Joyce et al. 2009: 74);

0. lateral branches of entoplastron more or less straight and merge anterior at a clear angle;
1. entoplastron wide and rounded, as seen in *P.* aff. *thomasii.*

75) Mobility of entoplastron and anterior development of hyoplastron (Joyce et al. 2009: 75); ORDERED

0. lateral branches of entoplastron abut loosely against hyoplastron, anterior rim of hyoplastron develops no anterior flap/shoulder;
1. lateral branches of entoplastron abut loosely against hyoplastron, but hyoplastron develops an anterior flap/shoulder, as seen in *Plastomenus* aff. *thomasii*;
2. entoplastron tightly integrated into anterior plastral lobe due to strong development of anterior flap/shoulder, as developed in *H. rememdium* and *H. arctochelys.*

76) Peripheral ossification (Joyce et al. 2009: 76);

0. lateral bridge ossification of plastron does not significantly extend beyond the lateral processes of hyo- and hypoplastron;
1. lateral bridge ossification of plastron extends laterally beyond bridge processes of the hyo- and hypoplastron and ossifies the peripheral aspects of the shell, as seen in *H. arctochelys.*

77) Number of lateral hyoplastral processes (Joyce and Lyson 2011: 77); ORDERED

1. one;
2. two;
3. three or more.

78) Extensive secondary palate consisting of infolded maxillae (Joyce and Lyson 2011: 78);

0. absent;
1. present, as developed in *Plastomenus thomasii.*

79) Accessory ridges of upper triturating surfaces (Joyce and Lyson 2011: 79);

0. absent;
1. present.

80) Posterior portion of narial canal defined by bone (Joyce and Lyson 2011: 80);

0. absent;
1. present, as developed in *Gilmoremys lancensis*.

81) Dentary symphysis (Joyce and Lyson 2011: 81);

0. short;
1. extremely long, mandible extremely elongate, as developed in *Plastomenus thomasii*.

82) Parietal contribution to orbits (Joyce and Lyson 2011: 82);

0. parietals neither contribute to orbit margins or orbit walls;
1. parietals either contribute to orbit walls, as developed in *Gilmoremys lancensis*, or orbit margin, as developed in *Plastomenus thomasii.*

83) Proportions of costals VIII (Joyce and Lyson 2011: 83);
0. wider than long to nearly square;
1. significantly taller than wide.

84) (New character): Ilium with expanded dorsal end;
0. present;
1. absent.

85) (New character): Hyoplastron with strongly serrated medial edge;
0. present;
1. absent.

86) (New character): Serrated medial edge of hyoplastron extends nearly to the posterior edge of bone ;
0. present;
1. absent.

87) (New character): Process on medial edge of hypoplastron;
0. of subequal size and radiating outward from the medial edge of the bone;
1. with enlarged anterior process separated by a gap from smaller posterior processes.

88) (New character): Ossification of basibranchials;
0. poorly ossified, especially posterior pair;
1. well ossified

List of homoplastic characters. Optimization onto the molecular topologyof Le et al. (2014) revealed that these characters are highly homoplastic. Of each character at least one state evolved minimally three times independently within crown-trionychids. Characters that are homoplastic across a large clade can still diagnose smaller clades and therefore we advise using implied weights instead of omitting them.

Width/length of nuchal bone (Meylan 1987: 1);

Number of plastral callosities (Meylan 1987: 9);

Pleurals (=costals) which meet at midline (Meylan 1987: 16);

Largest adult size 200 mm or less (disc length; Meylan 1987: 24);

Jugal contacts squamosal (Meylan 1987: 32);

Jugal contacts parietal on skull surface (Meylan 1987: 34);

Dorsal edge of aperture narium externum laterally emarginated (Meylan 1987: 41);

Vomer divides maxillae (Meylan 1987: 48);

Vomer reaches intermaxillary foramen (Meylan 1987: 49);

Basisphenoid shape (Meylan 1987: 64);

Epipterygoid, if present, contacts the palatine (Meylan 1987: 68);

Epipterygoid contacts prootic anterior to foramen nervi trigemini (Meylan 1987: 71);

Epipterygoid fuses to pterygoid (Meylan 1987: 73) ;

Postorbital bar relative to orbit (Meylan 1987: 75);

Quadratojugal participates in processus trochlearis oticum (Meylan 1987: 76);

Proportion of processus trochlearis oticum made up by parietal (Meylan 1987: 78);

Ventral keel on 8th cervical present and limited to posterior end (Meylan 1987: 87);

Number of ossifications in corpus hyoidis (Meylan 1987: 90);

Number of ossifications in comu branchiale II (Meylan 1987: 91);

Foramen intermandibularis caudalis enclosed by prearticular (Meylan 1987: 98);

Metischial processes present and distinct (Meylan 1987: 109);

Angle of acromion process to scapula approaches that of coracoid to acromion (Meylan 1987: 112);

Modifications of the matrix of Li et al. (2015) and Joyce et al. (2016).

Development of surface sculpturing of carapace and plastron (Joyce et al. 2009: 67).

Comment: An additional third character state was added to the previous two states in order to capture the sculpturing in Early Cretaceous pan-trionychids from Asia. The new state is as follows: 2. plastron sculpture greatly subdued or absent.

Suprascapular fontanelles (Meylan 1987: 18).

Perochelys lamadongensis: ?→3. The fontanelle is open after Li et al., (2015).

Number of neurals (fused 1 and 2 counted as 2; Meylan 1987: 14).

Perochelys lamadongensis: 3→1.

<u>Comment:</u> *Li et al. (2015) scores eight neural elements for this taxon but does not take into account that the first two of these are fused and therefore should be counted as separate elements following the original character of Meylan (1987). Since Li et al. (2015a) did not change the definition of the character, Perochelys lamadongensis scores as having nine neurals.*
Joyce et al. (2016) changed this character to: "Number of neurals: eight (1); seven (2); six or less (3)"and counts the fused neurals 1 and 2 as a single element. We return to the original version of the character because this will allow future works to address morphological variation in phylogenetic reconstruction of soft-shell turtles. Like Meylan (1987), we count the fused neurals 1 and 2 as two elements.

Epiplastron shape (Meylan 1987: 19);

Hutchemys sterea: 2→1
Hutchemys tetraneton: ?→1
Hutchemys arctochelys: 2→?
Hutchemys rememdium: 2→?

<u>Comment:</u> *Joyce and Lyson (2011) scored many plastomenids as having a J-shaped epiplastron that is otherwise only present in most cyclanorbines. TMP 93.94.1 of Hutchemys tetanetron reveals and figures of H. sterea (Hutchison 2009, figs.3-9) reveal that these taxa (and perhaps plastomenids in general) have J-shaped, rather than I-shaped epiplastron. Since no epiplastral elements have been reported for H. arctochelys or rememdium we rescore these taxa as unknown.*

Largest adult size 200 mm or less (disc length; Meylan 1987: 24);

Perochelys lamadongensis: *?→2*

<u>Comment:</u> *based on the degree of ossification, the only specimen of Perochelys lamadongensis was an adult individual and the carapace is about 100 mm long. This is in accordance with the estimated size of other Early Cretaceous trionychid fossils that are also considered to represent adults (i.e. "Petrochelys*

kyrgyzensis Nessov 1995, Perochelys hengshanensis sp. nov). "Trionyx" jixiensis Li et al. 2015b is slightly larger but still well under 200 mm (~150 mm).

Total number of peripherals (Meylan 1987: 5);

1. 22
2. 20
3. 14-18
4. 0

Comment: Joyce et al. (2016) changed the character to: "Neomorphic peripherals: absent (0); present (1)." However, there is no way to a priori exclude the homology of the ossicles in Lissemys punctata with the peripheral elements of turtles based on morphology or phylogeny (Delfino et al. 2014; Le et al. 2014). We therefore return to the version of the character that Meylan (1987) originally proposed.

Point of reversal of orientation of neurals (Meylan 1987: 17);

1. at neural eight
2. at neural seven
3. at neural six or seven
4. at neural six
5. at neural four, five, or six
6. (New state) no reversal

Comment: Joyce et al. (2016) changed this character to: "Point of neural reversal: neural VII (1); neural VI (2); neural V (3); neural IV (4)." We return to the original version of the character because this will allow future works to address morphological variation in the phylogenetic reconstruction of soft-shell turtles.

Jugal contacts parietal on skull surface (Meylan 1987: 34);

1. no
2. in one-half of sample
3. yes

Comment: Joyce et al. (2016) changed this character to: "Jugal-parietal contact absent (1); present (2)." We return to the original version of the character because this will allow future works to address morphological variation in the phylogenetic reconstruction of soft-shell turtles. Moreover, Meylan's character explicitly pertained to a contact on the skull surface.

Basisphenoid shape (Meylan 1987: 64);

1. not medially constricted
2. occasionally medially constricted
3. medially constricted

Comment: *Joyce et al. (2016) changed this character to: "Basisphenoid shape: not constricted (1); medially constricted (2)." We return to the original version of the character because this will allow future works to address morphological variation in the phylogenetic reconstruction of soft-shell turtles.*

Epipterygoid, if present, contacts the palatine (Meylan 1987: 68);

1. yes
2. in ca. 50%
3. no

Epipterygoid contacts prootic anterior to foramen nervi trigemini (Meylan 1987: 71);

1. no
2. in ca. 50%
3. yes

Comment: *Joyce et al. (2016) removed the second state from both these characters. We return to the original version of the character because this will allow future works to address morphological variation in the phylogenetic reconstruction of soft-shell turtles.*

Changes in the coding of *Aspideretoides foveatus*. Joyce et al. (2016) scored most cranial characters unknown but specimens at TMP (not studied by those authors) allow for a re-scoring of this species. Otherwise, we only have a few disagreements with the scorings of Joyce et al. (2016).

Dorsal edge of aperture narium externum medially emarginated (Meylan 1987: 42);

Joyce et al. (2016) scored it unknown but new material (TMP 81.24.7) shows that the aperture is emarginated laterally, but not medially.

Basisphenoid contacts palatines (Meylan 1987: 46);

Joyce et al. (2016) scored it unknown. TMP 2005.12.557 shows the contact.

Vomer contacts basisphenoid (Meylan 1987: 51);

Joyce et al. (2016) scored it unknown. TMP 2005.12.557 shows that the contact is absent.

Size of foramen palatinum posterius (Meylan 1987: 53);

Joyce et al. (2016) scored it unknown. TMP 2005.12.557 shows that the foramen is small.

Foramen palatinum posterius forms in (Meylan 1987: 54);

Joyce et al. (2016) scored it unknown. TMP 2005.12.557 shows that the foramen is small.

Maxilla contacts frontal in front of orbit (Meylan 1987: 62);

Joyce et al. (2016) scored it unknown. TMP 81.24.7 shows that the contact is absent.

Premaxilla absent (Meylan 1987: 65);

Joyce et al. (2016) scored it unknown. TMP 81.24.7 shows that the premaxilla is present.

Vomer lost (Meylan 1987: 66);

Joyce et al. (2016) scored it unknown. TMP 2005.12.557 shows that the premaxilla is present.

Jugal contacts orbit (Meylan 1987: 67);

Joyce et al. (2016) scored it unknown. TMP 2005.12.557 shows that the contact is present.

Postorbital bar relative to orbit (Meylan 1987: 75);

Joyce et al. (2016) scored it unknown. Based on TMP 2005.12.557 we score about equal to orbit to 1/3 of orbit (state 1).

Proportion of processus trochlearis oticum made up by parietal (Meylan 1987: 78);

1. 15.6% or less
2. 22.1% or more

Joyce et al. (2016) scored it unknown. Based on TMP 2005.12.557 we score state 1.

Number of ossifications in corpus hyoidis (Meylan 1987: 90);

TMP 81.24.7 shows that there are 6 ossifications (state 2).

Basihyals in close contact and projecting anteriorly (Meylan 1987: 93);

TMP 81.24.7 shows that the basihyals are not in close contact (state 1).

Dentary symphysis (Joyce and Lyson 2011: 81);

TMP 81.24.7 shows that the symphysis is considerably shorter than in Plastomenus thomasii.

Character Matrix

Carettochelys insculpta:

(12)-1-21121115?3-1-2?-11?11111211121211121111112?113-11111----122212200---01--0-00

000101—0

Cycloderma aubryi:

2121411122221211211112113221122142213111111222-12111111221121221211000000001

01000000?0111

Pelochelys bibroni:

3222411311121152112211113111111221112111311310212111122322111121220000000000

02000000?0101

Amyda cartilaginea:

3222411211111122132211111121122121112112111210213112121321121121120000000000

0200000010111

Cyclanorbis elegans:

2221411421221221112211113112122132213111111112-12211221121121212221000000001

01000000?011?

Rafetus euphraticus:

3222412411131252112211111121211121113112111110113212221331112122120000000000

02000000?0111

Apalone ferox:

32224123111332421122112111311111211131111111011321221133111112212000000{0

1}0{0 1}010000001011?

Nilssonia formosus:

22224113111111211122111231211221211131131111111131111---2112212---0000000000?

000000????1

Cycloderma frenatum:

211141112122122121111211321112214221311122132-122112112211212212110000000001

010000001011?

Nilssonia gangetica:

3221411211122231122211111211221211131131111111211211123112112112000000000

02000000?0111

Nilssonia hurum:

3221411211112231122211112131122121113113111112131111113111211211200000000

02000000????1

Chitra indica:

3232411311111131112211213211111211121112113103130011223221121212200000000

02000000?0101

Apalone mutica:

42224121111232431122212221311121211131111111 2-11222221122111121220000000{

0 1}01000000?011?

Lissemys punctata:

2111321122241221211112112111121122213111112111111111121121211212000000001

1100000011-11

Cyclanorbis senegalensis:

322142102125-4611111111132211111322131111113 2-121111111211212222210000000010

2000000?0111

Pelodiscus sinensis:

42224111111232421322211221311221221231131111121131121111221111121120000000000

0200000010111

Apalone spinifera:

32224121111332431122212111311111211131121111 2-11322221123111112122000000000{

0 1}0100000010111

Palea steindachneri:

22224113111212211322211131311221211231131111 2-3131211--221111-2---0000000000?0

00000?????

Dogania subplana:

42224113111120231322211121311221211232121111 1-112122111321121121120000000000

020000001011?

Trionyx triunguis:

3222411311131232112211111131122121113121111101121222112211111211200000000

0200000010101

Aspideretoides foveatus:

42314113111312411122 11????212??12????1?1111?????11?1??2??11??????10000001101?

??0?011-11

Gilmoremys lancensis:

42?1411312131261??2211?23231{12}22132223111?1112-3131111????????????0000000?1

02111110?1-1?

Plastomenus thomasii:

42?141121224-361?11111?11???12-122223211111??????-211??????1??????0000001110210

0111???-?

H. rememdium:

42?1411112231221??2111???21??11110011201????

?{0 1}?1--?

H. arctochelys:

42?1411112231221??1111???10111111211????

?1?1--?

H. tetraneton:

42?14113112312211??121???1101001?20?????

???????

H. sterea:

32?1411112231251111121??1001001120?????

?1?????

Perochelys lamadongensis:

421241231111?04{23}12?221?23?????????????????1???????1?1?12111??121??2000000000

2???000?0000

Perochelys hengshanensis:

?2??4??3111?????????21???????????????????????????????????????1?1??2??00000002?????

?0000?

Adocus lineolatus:

2-1-111-11-(45)12-1-1?-11?1111111-1(12)2111121111?12??3-11121----121112100--001--0-

01?00101--0

Gobiapalone orlovi:

42224122111212421222211?11?211111211(01)31?1?1???????221111????1?1211?000000000

020000001011?

Petrochelys kyrgyzensis:

42??41131111?14112?221??20000000002????

?0?000?

Nemegtemys conflata:

????????2??

???

Kuhnemys maortuensis:

????4?131111?14?1???11??0?00??0000??????

??000?

Molecular backbone constraint

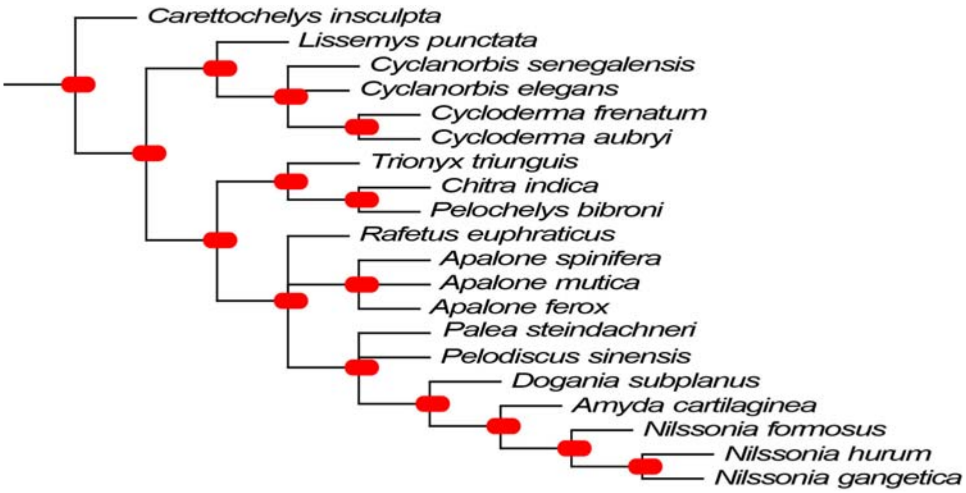

Figure S1. Molecular backbone constraint used in the parsimony analysis. This molecular backbone is the combined (“consensus”) phylogeny of Liebing et al. (2012), Le et al. (2014), and Li et al. (2017). Only nodes of at least 99 % bootstrap/posterior probability values were constrained.

Ontogenetic ossification of post-hatchling trionychids shell

The Early Cretaceous turtles from China are represented by a multiple individuals all of small size and all with a highly fenestrated, reduced carapace. According to Nessov (1995) *Petrochelys kyrgyzensis* is represented by the disarticulated remains of several individuals. In addition, *Perochelys lamadongensis*, “*Trionyx*” *jixiensis*, and the *Perochelys hengshanensis* are presented by one individual each. Nessov (1995) interpreted the specimens of *Petrochelys kyrgyzensis* to be from adult individuals. Since the other Early Cretaceous trionychids are morphologically similar, they would likely also be adults. To identify ossification patterns that

would allow this to be independently evaluated, the pattern of ossification of the trionychid shell in a series of post-hatchling idividuals was examined. Since *Dogania subplana* is most similar to the Early Cretaceous trionychids in the degree of fenestration of the plastron and it is well represented in museum collections by individuals of a variety of sizes and stages of development, it was used as a modern analogue. Four stages could be recognized on the basis of relative length of the free ends of the ribs on costals numbers three to five (Fig. S2) . In the least mature individuals, the length of these was greater than the length of the more proximal portion of the costal. In the second stage, the length of the free end of the rib was less than the more proximal ossified portion of the costal but greater than the antero-posterior width of the distal end of the costal. In the third, the length of the free end of the rib was less than the antero-posterior width of the distal end of the costal. In the fourth, the free ends of the ribs were no longer visible. Most of the individuals in museum collections were at stage 2 or stage 3. Individual at stage 2 was 60% size of the largest individuals observed. This variation in adult size is not unexpected in reptiles with indeterminate growth, and an individual of stage 2 size would be considered a small adult. The shape of the plastron of individuals of *Dogana subplana* at stage 2 differs little from that of an individual at stage 4 in the development of fenestrae and ossification of the plastral elements. Thus, based on the pattern of ossification of the shell of *Dogania subplana*, the Early Cretaceous trionychids from Asia can be assumed to have had an adult morphology, although possibly they are likely not representative of the maximum size reached by individual of their respective species.

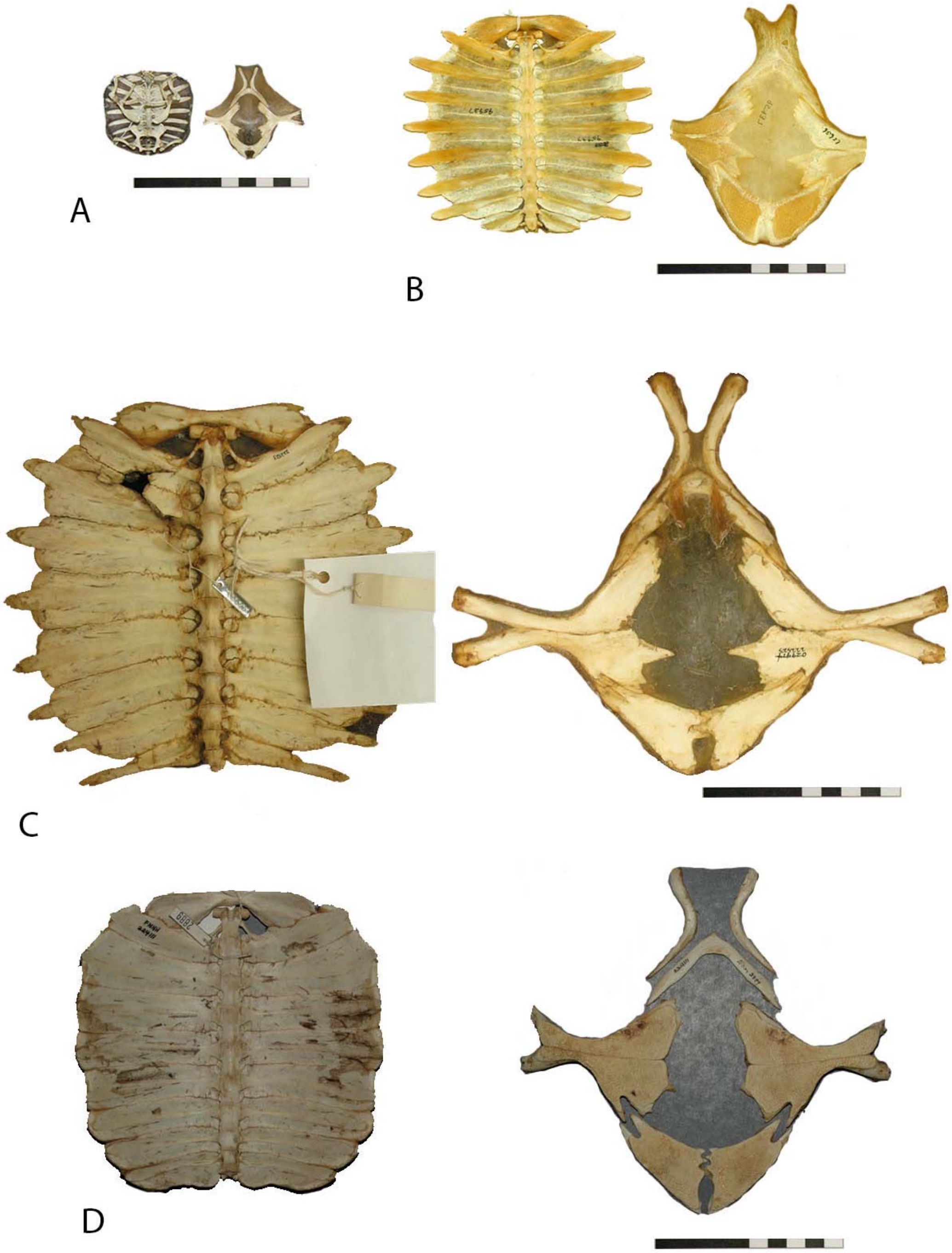

Figure S2. Post-hatchling ontogenetic series of shells of *Dogania suplana* showing ossification of the carapace. A) specimen NHMW 1870, inidivudal at stage 1 of development, free ends of the ribs longer than the more proximal ossified portion of the carapace. B) specimen UCMZ 95937, individual at stage 2 of development, free ends of the ribs shorter than the more proximal ossified portion of the carapace but longer than the antero-posterior

width of the lateral end of the costal. Lateral ends of hyo- and hypoplastron missing in this specimen. C) specimen USNM 222523, individual with the free ends of the ribs shorter than the antero-posterior width of the lateral end fo the carapace. D) specimen FMNH 224111, individual at stage 4 of development, free ends of ribs not distinct on lateral end of carapace. Images to scale, scale bar equals 10 cm.

Phylogenetic position of *Nemegtemys conflata*

Nemegtemys conflata, a fragmentary taxon from the Maastrichtian Nemegt Formation of Mongolia, was originally referred to the Cyclanorbinae by Danilov et al. (2014) on the basis of fusion of the hyo- and hypoplstron in an individual of small size. Although it is represented by two plastral fragments only (on a partial fused hyo-hypoplstron), this position is supported by our phylogenetic analysis (Fig. S3).

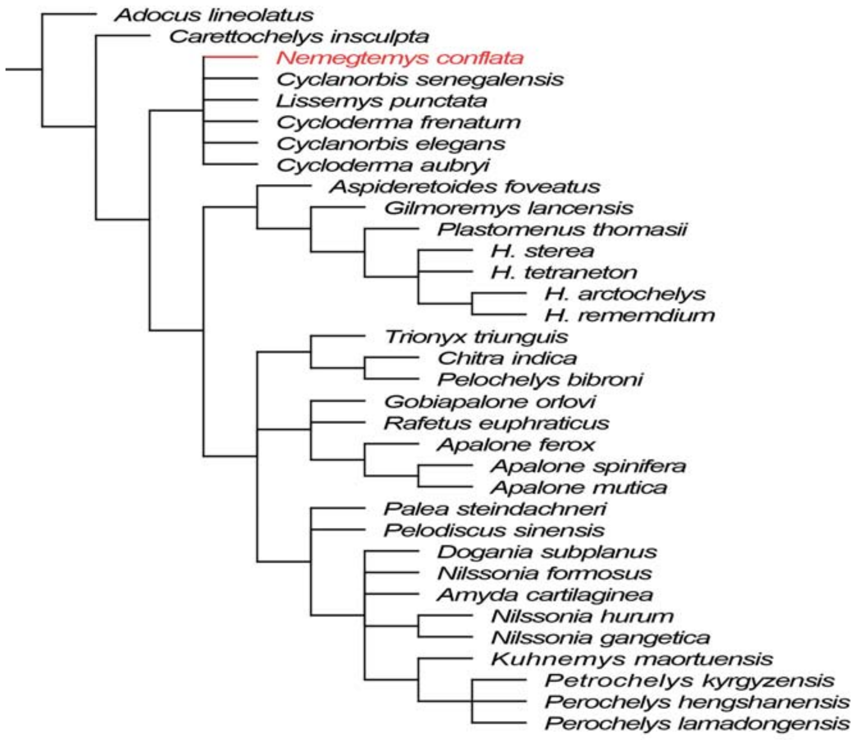

Figure. S3. Strict consensus of 179 trees including *Nemegtemys conflata* using equal weighting. Implied weighting results in the same position of this taxon.

Common synapomorphies to 6 trees under implied weighting (K value = 3).

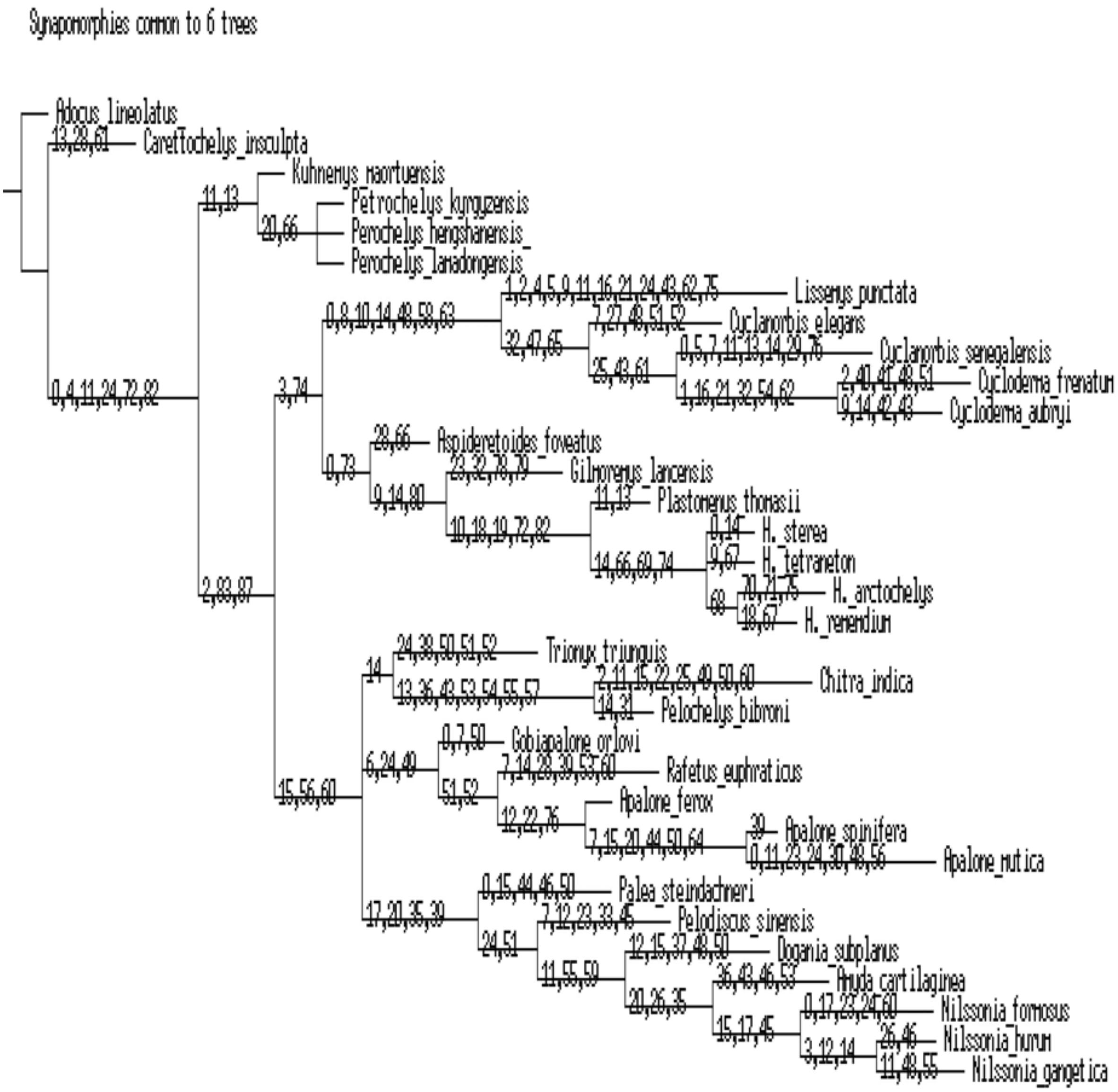

(Node numbers refer to nodes in consensus)

Carettochelys insculpta:

All trees:

Pleurals which meet at midline (13): seventh and eighth or eighth only --> sixth, seventh, and eighth or seventh and eighth

Basisphenoid contacts palatines (28): no --> yes

Ilia curve medially (61): no --> yes

Cycloderma aubryi:

All trees:

Fusion of xiphiplastra (9): absent --> present
Point of reversal of orientation of neurals (14): at neural seven --> at neural eight
Jugal contacts orbit (42): yes --> no
Epipterygoid, if present, contacts the palatine (43): no --> in ca. 50%

Pelochelys bibroni:
All trees:
Point of reversal of orientation of neurals (14): at neural six or seven --> at neural four, five, or six
Vomer contacts basisphenoid (31): no --> occasionally

Amyda cartilaginea:
All trees:
Foramen posterius canalis carotici interni relative to lateral crest of basioccipital tubercle (36): below --> in it
Epipterygoid, if present, contacts the palatine (43): yes --> in ca. 50%
Epipterygoid contacts prootic anterior to foramen nervi trigemini (46): no --> in ca. 50%
Ventral keel on 8th cervical present and limited to posterior end (53): no --> yes

Cyclanorbis elegans:
All trees:
Number of plastral callosities (7): seven, or five, or four --> two
Dorsal edge of aperture narium externum medially emarginated (27): no --> yes
Epipterygoid fuses to pterygoid (48): in subadults --> in adults only
Quadratojugal participates in processus trochlearis oticum (51): no --> yes
Proportion of processus trochlearis oticum made up by parietal (52): 15.6% or less --> 22.1% or more

Rafetus euphraticus:
All trees:
Number of plastral callosities (7): four --> two
Point of reversal of orientation of neurals (14): at neural six --> at neural four, five, or six
Basisphenoid contacts palatines (28): no --> yes
Basisphenoid shape (39): not medially constricted --> occasionally medially constricted
Ventral keel on 8th cervical present and limited to posterior end (53): no --> yes
Foramen intermandibularis caudalis enclosed by prearticular (60): sometimes --> never

Apalone ferox:

All trees:

No autapomorphies:

Nilssonia formosus:

All trees:

Width-length of nuchal bone (0): greater than 3 --> greater than 2

Length epiplastra anterior to entoplastron contact (17): intermediate --> short

Jugal contacts squamosal (23): no --> in one half of sample

Jugal contacts parietal on skull surface (24): no, or in one half of sample --> yes

Foramen intermandibularis caudalis enclosed by prearticular (60): sometimes --> never

Cycloderma frenatum:

All trees:

Position of anterior edge of first body vertebra relative to nuchal bone (2): middle of nuchal --> posterior edge of nuchal

Premaxilla absent (40): no --> occasionally

Vomer lost (41): no --> yes

Epipterygoid fuses to pterygoid (48): in subadults --> in adults only

Quadratojugal participates in processus trochlearis oticum (51): no --> yes

Nilssonia gangetica:

All trees:

Number of neurals (fused 1 and 2 counted as two) (11): nine --> eight or nine

Epipterygoid fuses to pterygoid (48): never --> in adults only

Number of ossifications in corpus hyoidis (55): 3 --> 8

Nilssonia hurum:

All trees:

Dorsal edge of aperture narium externum laterally emarginated (26): weakly --> strongly

Epipterygoid contacts prootic anterior to foramen nervi trigemini (46): no --> in ca. 50%

Chitra indica:

All trees:

Position of anterior edge of first body vertebra relative to nuchal bone (2): middle of nuchal --> anterior edge of nuchal

Number of neurals (fused 1 and 2 counted as two) (11): eight or nine --> nine

Suprascapular fontanelles (15): closed in large adults only --> closed at hatching

Sexual dimorphism in disc length (22): no --> yes

Vomer contacts prefrontal (25): yes --> no

Average ratio of intermaxillary foramen length to length primary palate (49): 0.20 to 0.40 --> 0.07

Postorbital bar relative to orbit (50): about equal to orbit to 1/3 of orbit --> about 2 times orbit diameter

Foramen intermandibularis caudalis enclosed by prearticular (60): sometimes --> never

Apalone mutica:

All trees:

Width-length of nuchal bone (0): greater than 3 --> greater than 4

Number of neurals (fused 1 and 2 counted as two) (11): eight --> eight or nine

Jugal contacts squamosal (23): no --> in one half of sample

Jugal contacts parietal on skull surface (24): no --> in one half of sample

Vomer reaches intermaxillary foramen (30): yes --> no

Epipterygoid fuses to pterygoid (48): never --> in adults only

Number of ossifications in comu branchiale II (56): 7 or more --> 2-6

Lissemys punctata:

All trees:

Anterior and posterior costiform processes of nuchal bone united (1): yes --> no

Position of anterior edge of first body vertebra relative to nuchal bone (2): middle of nuchal --> posterior edge of nuchal

Total number of peripherals (4): 0 --> 14-18

Prenuchal bone (5): absent --> present

Fusion of xiphiplastra (9): absent --> present

Number of neurals (fused 1 and 2 counted as two) (11): eight or nine, or eight --> seven or eight

Epiplastron shape (16): J-shaped --> I-shaped

Carapace margin straight to concave posteriolaterally (21): no --> yes

Jugal contacts parietal on skull surface (24): yes --> in one half of sample

Epipterygoid, if present, contacts the palatine (43): yes --> in ca. 50%

Ischia extend into thyroid fenestra (62): no --> yes

Peripheral ossification (75): lateral bridge ossification of plastron does not significantly extend beyond the lateral processes of hyo- and hypoplastron --> lateral bridge ossification of plastron extends laterally beyond bridge processes of the hyo- and hypoplastron and ossifies the peripheral aspects of the shell, as seen in *H. arctochelys*

Cyclanorbis senegalensis:

All trees:

Width-length of nuchal bone (0): greater than 2 --> greater than 3

Prenuchal bone (5): absent --> present

Number of plastral callosities (7): seven --> nine

Number of neurals (fused 1 and 2 counted as two) (11): eight or nine, or eight --> seven or fewer

Pleurals which meet at midline (13): seventh and eighth or eighth only --> more than sixth, seventh, and eighth

Point of reversal of orientation of neurals (14): at neural seven --> no reversal

Vomer divides maxillae (29): no --> yes

Number of lateral hyoplastral processes (76): one --> two

Pelodiscus sinensis:

All trees:

Number of plastral callosities (7): five, or four --> seven

Variability in position of neural reversal (12): always at same neural --> highly variable

Jugal contacts squamosal (23): no --> in one half of sample

Foramen palatinum posterius forms in (33): palatine and pterygoid and/or maxilla --> palatine only

When epipterygoid is present pterygoid contacts foramen nervi trigemini (45): between epipterygoid and quadrate or not at all --> between epipterygoid and parietalor not at all

Apalone spinifera:

All trees:

Basisphenoid shape (39): not medially constricted --> occasionally medially constricted

Palea steindachneri:

All trees:

Width-length of nuchal bone (0): greater than 3 --> greater than 2

Suprascapular fontanelles (15): closed in large adults only --> closed at hatching

Contact between pterygoid and foramen nervi trigemini occurs when epipterygoid is present (44): yes --> no

Epipterygoid contacts prootic anterior to foramen nervi trigemini (46): no --> yes

Postorbital bar relative to orbit (50): about equal to orbit to 1/3 of orbit --> less than 1/5 of orbit

Dogania subplanus:

All trees:

Variability in position of neural reversal (12): always at same neural --> always at adjacent neurals

Suprascapular fontanelles (15): closed in large adults only --> open throughout life

Maxilla contacts frontal in front of orbit (37): no --> yes

Epipterygoid fuses to pterygoid (48): never --> in adults only
Postorbital bar relative to orbit (50): about equal to orbit to 1/3 of orbit --> less than 1/5 of orbit

Trionyx triunguis:
All trees:
Jugal contacts parietal on skull surface (24): yes --> no
Exoccipital contacts pterygoid (38): no --> yes
Postorbital bar relative to orbit (50): about equal to orbit to 1/3 of orbit --> less than 1/5 of orbit
Quadratojugal participates in processus trochlearis oticum (51): no --> yes
Proportion of processus trochlearis oticum made up by parietal (52): 15.6% or less --> 22.1% or more

Aspideretoides foveatus:
All trees:
Basisphenoid contacts palatines (28): no --> yes
Development of surface sculpturing of carapace and plastron (66): all metaplastic portions of carapace and plastron have trionychid sculpturing --> trionychid pattern grades towards the center of carapacial and plastral disk to a smooth pattern, as developed in *Hutchemys rememdium* and *Hutchemys arctochelys*

Gilmoremys lancensis:
All trees:
Jugal contacts squamosal (23): no --> in one half of sample
Size of foramen palatinum posterius (32): small --> small and divided
Accessory ridges of upper triturating surfaces (78): absent --> present
Posterior portion of narial canal defined by bone (79): absent --> present, as developed in *Gilmoremys lancensis*

Plastomenus thomasii:
All trees:
Number of neurals (fused 1 and 2 counted as two) (11): eight --> seven or eight
Pleurals which meet at midline (13): seventh and eighth or eighth only --> sixth, seventh, and eighth or seventh and eighth

H. rememdium:
All trees:
Depressions on eighth pleurals for contact of ilia (18): present --> absent
Nuchal notch (67): anterior rim of nuchal convex or slightly notched --> anterior rim of carapace with deep nuchal notch, as developed in *H. rememdium*

H. arctochelys:

All trees:

Lateral notch in carapace at the level of costal 5 (70): absent, lateral carapacial margin rounded --> present, lateral carapacial margin shows a waist, as developed in *H. arctochelys*

Skin callosity developed on the visceral side of costals 6 and 7 (71): absent, visceral side smooth --> present, visceral side sometimes develops a callosity, as seen in *H. arctochelys*

Peripheral ossification (75): lateral bridge ossification of plastron does not significantly extend beyond the lateral processes of hyo- and hypoplastron --> lateral bridge ossification of plastron extends laterally beyond bridge processes of the hyo- and hypoplastron and ossifies the peripheral aspects of the shell, as seen in *H. arctochelys*

H. tetraneton:

All trees:

Fusion of xiphiplastra (9): present --> absent

Nuchal notch (67): anterior rim of nuchal convex or slightly notched --> anterior rim of carapace with deep nuchal notch, as developed in *H. rememdium*

Some trees:

Number of plastral callosities (7): seven, or five --> four

H. sterea:

All trees:

Width-length of nuchal bone (0): greater than 4 --> greater than 3

Point of reversal of orientation of neurals (14): at neural seven --> at neural four, five, or six

Perochelys lamadongensis:

Some trees:

Size of eighth pleurals (6): large --> reduced or absent

Pleurals which meet at midline (13): eighth only --> none

Suprascapular fontanelles (15): closed at hatching --> closed in large adults only, or open throughout life

Perochelys hengshanensis:

All trees:

No autapomorphies:

Adocus lineolatus:

All trees:

No autapomorphies:

Gobiapalone orlovi:

All trees:

Width-length of nuchal bone (0): greater than 3 --> greater than 4

Number of plastral callosities (7): four --> five

Postorbital bar relative to orbit (50): about equal to orbit to 1/3 of orbit --> less than 1/5 of orbit

Petrochelys kyrgyzensis:

All trees:

No autapomorphies:

Kuhnemys maortuensis:

All trees:

No autapomorphies:

Node 35:

All trees:

No synapomorphies

Node 36:

All trees:

Anterior and posterior costiform processes of nuchal bone united (1): yes --> no

Epiplastron shape (16): J-shaped --> I-shaped

Carapace margin straight to concave posteriolaterally (21): no --> yes

Size of foramen palatinum posterius (32): small and divided --> many small openings

Strong dorsal processes on cervicals (54): no --> yes

Ischia extend into thyroid fenestra (62): no --> yes

Node 37:

All trees:

Vomer contacts prefrontal (25): yes --> no

Epipterygoid, if present, contacts the palatine (43): yes --> no

Ilia curve medially (61): no --> yes

Node 38:

All trees:

Size of foramen palatinum posterius (32): small --> small and divided

Epipterygoid contacts prootic posterior to foramen nervi trigemini (47): no --> yes

Coracoid longest of three pectoral processes (65): yes --> no

Node 39:

All trees:

Width-length of nuchal bone (0): greater than 3 --> greater than 2

Hyoplastra and hypoplastra fuse just after hatching (8): no --> yes

Hypo-xiphiplastral union (10): xiphiplastra lateral to hypoplastra --> hypoplastra lateral to xiphiplastra

Point of reversal of orientation of neurals (14): at neural six --> at neural seven

Epipterygoid fuses to pterygoid (48): never --> in subadults

Basihyals in close contact and projecting anteriorly (58): no --> yes

Metischial processes present and distinct (63): yes --> no

Node 40:

All trees:

First and second neurals fused (3): yes --> no

Mobility of entoplastron and anterior development of hyoplastron (74): lateral branches of entoplastron abut loosely against hyoplastron, anterior rim of hyoplastron develops no anterior flap/shoulder --> lateral branches of entoplastron abut loosely against hyoplastron, but hyoplastron develops an anterior flap/shoulder, as seen in *Plastomenus* aff. *thomasii*

Node 41:

All trees:

Position of anterior edge of first body vertebra relative to nuchal bone (2): posterior edge of nuchal --> middle of nuchal

Ilium with expanded dorsal end (83): present --> absent

Ossification of basibranchials (87): poorly ossified, especially posterior pair --> well ossified

Node 42:

All trees:

Width-length of nuchal bone (0): greater than 2 --> greater than 3

Total number of peripherals (4): 20 --> 0

Number of neurals (fused 1 and 2 counted as two) (11): seven or eight, or seven or fewer --> eight or nine, or eight

Jugal contacts parietal on skull surface (24): no --> yes

Mid-line contact of hyoplastra, hypoplastra and xiphiplastra (72): hyo-, hypo and xiphiplastra contact another fully along the entire mid-line in adults, as developed in *H. rememdium* and *H. arctochelys* --> hyo-, hypo- and xiphiplastra do not contact another fully, even in adults

Proportions of costals VIII (82): significantly taller than wide --> wider than long to nearly square

Node 43:

All trees:

Pleurals which meet at midline (13): seventh and eighth or eighth only --> eighth only

Foramen posterius canalis carotici interni relative to lateral crest of basioccipital tubercle (36): below --> in it

Epipterygoid, if present, contacts the palatine (43): yes --> no

Ventral keel on 8th cervical present and limited to posterior end (53): no --> yes

Strong dorsal processes on cervicals (54): no --> yes

Number of ossifications in corpus hyoidis (55): 8 --> 3

Ossifications of comu branchiale II broad and strongly sutured (57): no --> yes

Node 44:

All trees:

Point of reversal of orientation of neurals (14): at neural six --> at neural six or seven

Node 45:

All trees:

Suprascapular fontanelles (15): closed at hatching --> closed in large adults only

Number of ossifications in comu branchiale II (56): 1 only --> 2-6

Foramen intermandibularis caudalis enclosed by prearticular (60): never --> sometimes

Node 46:

All trees:

Largest adult size 200 mm or less (disc length) (20): yes --> no

Dorsal edge of aperture narium externum laterally emarginated (26): strongly --> weakly

Enclosed foramen jugulare posterius (35): enclosed by opisthotic --> enclosed by pterygoid

Node 47:

All trees:

Number of neurals (fused 1 and 2 counted as two) (11): eight or nine --> nine

Number of ossifications in corpus hyoidis (55): 8 --> 3

Symphyseal ridge strong and present in a depression (59): no --> yes

Node 48:

All trees:

Jugal contacts parietal on skull surface (24): yes --> in one half of sample

Quadratojugal participates in processus trochlearis oticum (51): no --> yes

Node 49:

All trees:

Length epiplastra anterior to entoplastron contact (17): short, or intermediate --> long

Largest adult size 200 mm or less (disc length) (20): no --> yes

Enclosed foramen jugulare posterius (35): enclosed by pterygoid --> enclosed by opisthotic

Basisphenoid shape (39): not medially constricted --> medially constricted

Node 50:

All trees:

Quadratojugal participates in processus trochlearis oticum (51): no --> yes

Proportion of processus trochlearis oticum made up by parietal (52): 15.6% or less --> 22.1% or more

Node 51:

All trees:

Size of eighth pleurals (6): large --> reduced or absent

Jugal contacts parietal on skull surface (24): yes --> no

Average ratio of intermaxillary foramen length to length primary palate (49): 0.20 to 0.40 --> about 0.60

Node 52:

All trees:

Variability in position of neural reversal (12): always at same neural --> highly variable

Sexual dimorphism in disc length (22): no --> yes

Number of lateral hyoplastral processes (76): two --> one

Node 53:

All trees:

Suprascapular fontanelles (15): closed in large adults only --> closed at hatching

Length epiplastra anterior to entoplastron contact (17): long --> intermediate

When epipterygoid is present pterygoid contacts foramen nervi trigemini (45): between epipterygoid and quadrate or not at all --> between prootic and epipterygoid or not at all

Node 54:

All trees:

First and second neurals fused (3): yes --> no

Variability in position of neural reversal (12): always at same neural --> always at adjacent neurals

Point of reversal of orientation of neurals (14): at neural seven --> at neural six or seven

Node 55:

All trees:

Number of plastral callosities (7): four --> seven

Suprascapular fontanelles (15): closed in large adults only --> open throughout life

Largest adult size 200 mm or less (disc length) (20): no --> yes

Contact between pterygoid and foramen nervi trigemini occurs when epipterygoid is present (44): yes --> no

Postorbital bar relative to orbit (50): about equal to orbit to 1/3 of orbit --> less than 1/5 of orbit

Angle of acromion process to scapula approaches that of coracoid to acromion (64): no --> yes

Node 56:

All trees:

Width-length of nuchal bone (0): greater than 3 --> greater than 4

Shape of deep portion of entoplastron (73): lateral branches of entoplastron more or less straight and merge anterior at a clear angle --> entoplastron wide and rounded, as seen in *P*. aff. *thomasii*

Node 57:

All trees:

Fusion of xiphiplastra (9): absent --> present

Point of reversal of orientation of neurals (14): at neural six --> no reversal

Dentary symphysis (80): short --> extremely long, mandible extremely elongate, as developed in *Plastomenus thomasii*

Node 58:

All trees:

Hypo-xiphiplastral union (10): xiphiplastra lateral to hypoplastra --> hypoplastra lateral to xiphiplastra

Depressions on eighth pleurals for contact of ilia (18): absent --> present

Bridge length (19): short --> long

Mid-line contact of hyoplastra, hypoplastra and xiphiplastra (72): hyo-, hypo- and xiphiplastra do not contact another fully, even in adults --> hyo-, hypo and xiphiplastra contact another fully along the entire mid-line in adults, as developed in *H. rememdium* and *H. arctochelys*

Proportions of costals VIII (82): wider than long to nearly square --> significantly taller than wide

Some trees:

Number of plastral callosities (7): four --> five

Node 59:

All trees:

Shape of neural 1 and 2 (neural 2 and 3 of Meylan 1987) (68): neurals 1 and 2 hexagonal with short posterior sides --> neural 1 circular to rectangular and neural 2 octagonal, as developed in *H. rememdium* and *H. arctochelys*

Node 60:

All trees:

Point of reversal of orientation of neurals (14): no reversal --> at neural seven

Development of surface sculpturing of carapace and plastron (66): all metaplastic portions of carapace and plastron have trionychid sculpturing --> trionychid pattern grades towards the center of carapacial and plastral disk to a smooth pattern, as developed in *Hutchemys rememdium* and *Hutchemys arctochelys*

Splitting of costals along distal margin (69): costal rim rounded or graded --> dorsal rims split into separately protruding dorsal and visceral portions, as developed in *H. rememdium* and *H. arctochelys*

Mobility of entoplastron and anterior development of hyoplastron (74): lateral branches of entoplastron abut loosely against hyoplastron, but hyoplastron develops an anterior flap/shoulder, as seen in *Plastomenus* aff. *thomasii* --> entoplastron tightly integrated into anterior plastral lobe due to strong development of anterior flap/shoulder, as developed in *H. rememdium* and *H. arctochelys*

Node 61:

All trees:

Largest adult size 200 mm or less (disc length) (20): no --> yes

Development of surface sculpturing of carapace and plastron (66): all metaplastic portions of carapace and plastron have trionychid sculpturing --> plastron sculpture greatly subdued or absent

Node 62:

All trees:

Number of neurals (fused 1 and 2 counted as two) (11): eight or nine, or eight --> nine

Pleurals which meet at midline (13): seventh and eighth or eighth only --> eighth only

Synapomorphies common to 24 trees, equal weighting

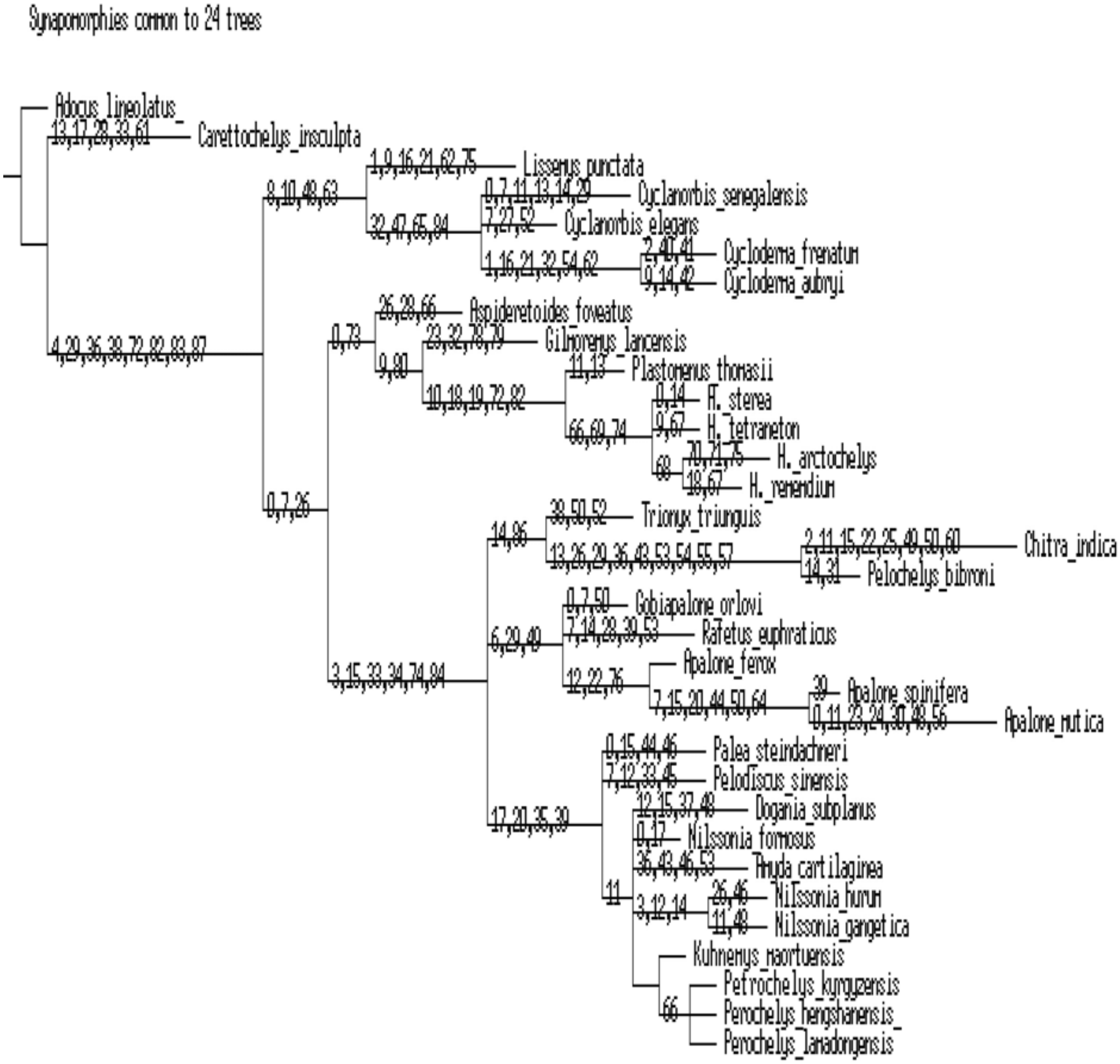

(Node numbers refer to nodes in consensus)

Carettochelys insculpta:

All trees:

- Pleurals which meet at midline (13): seventh and eighth or eighth only --> sixth, seventh, and eighth or seventh and eighth
- Length epiplastra anterior to entoplastron contact (17): short --> intermediate
- Basisphenoid contacts palatines (28): no --> yes
- Foramen palatinum posterius forms in (33): palatine only --> palatine and pterygoid and/or maxilla

Ilia curve medially (61): no --> yes

Cycloderma aubryi:

All trees:

Fusion of xiphiplastra (9): absent --> present

Point of reversal of orientation of neurals (14): at neural seven --> at neural eight

Jugal contacts orbit (42): yes --> no

Some trees:

Dorsal edge of aperture narium externum laterally emarginated (26): no --> weakly

Epipterygoid, if present, contacts the palatine (43): no --> in ca. 50%

Pelochelys bibroni:

All trees:

Point of reversal of orientation of neurals (14): at neural six or seven --> at neural four, five, or six

Vomer contacts basisphenoid (31): no --> occasionally

Amyda cartilaginea:

All trees:

Foramen posterius canalis carotici interni relative to lateral crest of basioccipital tubercle (36): below --> in it

Epipterygoid, if present, contacts the palatine (43): yes --> in ca. 50%

Epipterygoid contacts prootic anterior to foramen nervi trigemini (46): no --> in ca. 50%

Ventral keel on 8th cervical present and limited to posterior end (53): no --> yes

Cyclanorbis elegans:

All trees:

Number of plastral callosities (7): seven --> two

Dorsal edge of aperture narium externum medially emarginated (27): no --> yes

Proportion of processus trochlearis oticum made up by parietal (52): 15.6% or less --> 22.1% or more

Some trees:

Depressions on eighth pleurals for contact of ilia (18): present --> absent

Bridge length (19): long --> short

Epipterygoid fuses to pterygoid (48): in subadults --> in adults only

Quadratojugal participates in processus trochlearis oticum (51): no --> yes

Rafetus euphraticus:

All trees:

Number of plastral callosities (7): four --> two

- Point of reversal of orientation of neurals (14): at neural six --> at neural four, five, or six
- Basisphenoid contacts palatines (28): no --> yes
- Basisphenoid shape (39): not medially constricted --> occasionally medially constricted
- Ventral keel on 8th cervical present and limited to posterior end (53): no --> yes

Some trees:

- Foramen intermandibularis caudalis enclosed by prearticular (60): sometimes --> never
- Metischial processes present and distinct (63): yes --> no

Apalone ferox:

Some trees:

- Metischial processes present and distinct (63): yes --> no

Nilssonia formosus:

All trees:

- Width-length of nuchal bone (0): greater than 3 --> greater than 2
- Length epiplastra anterior to entoplastron contact (17): intermediate --> short

Some trees:

- Jugal contacts squamosal (23): no --> in one half of sample
- Foramen intermandibularis caudalis enclosed by prearticular (60): sometimes --> never

Cycloderma frenatum:

All trees:

- Position of anterior edge of first body vertebra relative to nuchal bone (2): middle of nuchal --> posterior edge of nuchal
- Premaxilla absent (40): no --> occasionally
- Vomer lost (41): no --> yes

Some trees:

- Epipterygoid fuses to pterygoid (48): in subadults --> in adults only
- Quadratojugal participates in processus trochlearis oticum (51): no --> yes

Nilssonia gangetica:

All trees:

- Number of neurals (fused 1 and 2 counted as two) (11): nine --> eight or nine
- Epipterygoid fuses to pterygoid (48): never --> in adults only

Some trees:

- Number of ossifications in corpus hyoidis (55): 3 --> 8

Nilssonia hurum:

All trees:

Dorsal edge of aperture narium externum laterally emarginated (26): weakly --> strongly

Epipterygoid contacts prootic anterior to foramen nervi trigemini (46): no --> in ca. 50%

Chitra indica:

All trees:

Position of anterior edge of first body vertebra relative to nuchal bone (2): middle of nuchal --> anterior edge of nuchal

Number of neurals (fused 1 and 2 counted as two) (11): eight or nine --> nine

Suprascapular fontanelles (15): closed in large adults only --> closed at hatching

Sexual dimorphism in disc length (22): no --> yes

Vomer contacts prefrontal (25): yes --> no

Average ratio of intermaxillary foramen length to length primary palate (49): 0.20 to 0.40 --> 0.07

Postorbital bar relative to orbit (50): about equal to orbit to 1/3 of orbit --> about 2 times orbit diameter

Foramen intermandibularis caudalis enclosed by prearticular (60): sometimes --> never

Apalone mutica:

All trees:

Width-length of nuchal bone (0): greater than 3 --> greater than 4

Number of neurals (fused 1 and 2 counted as two) (11): eight --> eight or nine

Jugal contacts squamosal (23): no --> in one half of sample

Jugal contacts parietal on skull surface (24): no --> in one half of sample

Vomer reaches intermaxillary foramen (30): yes --> no

Epipterygoid fuses to pterygoid (48): never --> in adults only

Number of ossifications in comu branchiale II (56): 7 or more --> 2-6

Lissemys punctata:

All trees:

Anterior and posterior costiform processes of nuchal bone united (1): yes --> no

Fusion of xiphiplastra (9): absent --> present

Epiplastron shape (16): J-shaped --> I-shaped

Carapace margin straight to concave posteriolaterally (21): no --> yes

Ischia extend into thyroid fenestra (62): no --> yes

Peripheral ossification (75): lateral bridge ossification of plastron does not significantly extend beyond the lateral processes of hyo- and hypoplastron --> lateral bridge ossification of plastron extends laterally beyond bridge processes of

the hyo- and hypoplastron and ossifies the peripheral aspects of the shell, as seen in *H. arctochelys*

Some trees:

Prenuchal bone (5): absent --> present

Epipterygoid, if present, contacts the palatine (43): yes --> in ca. 50%

Cyclanorbis senegalensis:

All trees:

Width-length of nuchal bone (0): greater than 2 --> greater than 3

Number of plastral callosities (7): seven --> nine

Number of neurals (fused 1 and 2 counted as two) (11): eight or nine, or eight, or seven or eight --> seven or fewer

Pleurals which meet at midline (13): seventh and eighth or eighth only --> more than sixth, seventh, and eighth

Point of reversal of orientation of neurals (14): at neural seven --> no reversal

Vomer divides maxillae (29): no --> yes

Some trees:

Prenuchal bone (5): absent --> present

Dorsal edge of aperture narium externum laterally emarginated (26): no --> weakly

Number of lateral hyoplastral processes (76): one --> two

Pelodiscus sinensis:

All trees:

Number of plastral callosities (7): five, or four --> seven

Variability in position of neural reversal (12): always at same neural --> highly variable

Foramen palatinum posterius forms in (33): palatine and pterygoid and/or maxilla --> palatine only

When epipterygoid is present pterygoid contacts foramen nervi trigemini (45): between epipterygoid and quadrate or not at all --> between epipterygoid and parietalor not at all

Some trees:

Width-length of nuchal bone (0): greater than 3 --> greater than 4

Jugal contacts squamosal (23): no --> in one half of sample

Apalone spinifera:

All trees:

Basisphenoid shape (39): not medially constricted --> occasionally medially constricted

Palea steindachneri:

All trees:

- Width-length of nuchal bone (0): greater than 3 --> greater than 2
- Suprascapular fontanelles (15): closed in large adults only --> closed at hatching
- Contact between pterygoid and foramen nervi trigemini occurs when epipterygoid is present (44): yes --> no
- Epipterygoid contacts prootic anterior to foramen nervi trigemini (46): no --> yes

Some trees:

- Point of reversal of orientation of neurals (14): at neural six --> at neural seven
- Jugal contacts parietal on skull surface (24): no, or in one half of sample --> yes
- Postorbital bar relative to orbit (50): about equal to orbit to 1/3 of orbit --> less than 1/5 of orbit
- Quadratojugal participates in processus trochlearis oticum (51): yes --> no

Dogania subplanus:

All trees:

- Variability in position of neural reversal (12): always at same neural --> always at adjacent neurals
- Suprascapular fontanelles (15): closed in large adults only --> open throughout life
- Maxilla contacts frontal in front of orbit (37): no --> yes
- Epipterygoid fuses to pterygoid (48): never --> in adults only

Some trees:

- Width-length of nuchal bone (0): greater than 3 --> greater than 4
- Pleurals which meet at midline (13): eighth only --> none
- Postorbital bar relative to orbit (50): about equal to orbit to 1/3 of orbit --> less than 1/5 of orbit

Trionyx triunguis:

All trees:

- Exoccipital contacts pterygoid (38): no --> yes
- Postorbital bar relative to orbit (50): about equal to orbit to 1/3 of orbit --> less than 1/5 of orbit
- Proportion of processus trochlearis oticum made up by parietal (52): 15.6% or less --> 22.1% or more

Some trees:

- Quadratojugal participates in processus trochlearis oticum (51): no --> yes

Aspideretoides foveatus:

All trees:

- Dorsal edge of aperture narium externum laterally emarginated (26): strongly --> weakly
- Basisphenoid contacts palatines (28): no --> yes
- Development of surface sculpturing of carapace and plastron (66): all metaplastic portions of carapace and plastron have trionychid sculpturing --> trionychid

pattern grades towards the center of carapacial and plastral disk to a smooth pattern, as developed in *Hutchemys rememdium* and *Hutchemys arctochelys*

Gilmoremys lancensis:

All trees:

Jugal contacts squamosal (23): no --> in one half of sample

Size of foramen palatinum posterius (32): small --> small and divided

Accessory ridges of upper triturating surfaces (78): absent --> present

Posterior portion of narial canal defined by bone (79): absent --> present, as developed in *Gilmoremys lancensis*

Some trees:

Jugal contacts parietal on skull surface (24): no --> yes

Plastomenus thomasii:

All trees:

Number of neurals (fused 1 and 2 counted as two) (11): eight --> seven or eight

Pleurals which meet at midline (13): seventh and eighth or eighth only --> sixth, seventh, and eighth or seventh and eighth

H. rememdium:

All trees:

Depressions on eighth pleurals for contact of ilia (18): present --> absent

Nuchal notch (67): anterior rim of nuchal convex or slightly notched --> anterior rim of carapace with deep nuchal notch, as developed in *H. rememdium*

H. arctochelys:

All trees:

Lateral notch in carapace at the level of costal 5 (70): absent, lateral carapacial margin rounded --> present, lateral carapacial margin shows a waist, as developed in *H. arctochelys*

Skin callosity developed on the visceral side of costals 6 and 7 (71): absent, visceral side smooth --> present, visceral side sometimes develops a callosity, as seen in *H. arctochelys*

Peripheral ossification (75): lateral bridge ossification of plastron does not significantly extend beyond the lateral processes of hyo- and hypoplastron --> lateral bridge ossification of plastron extends laterally beyond bridge processes of the hyo- and hypoplastron and ossifies the peripheral aspects of the shell, as seen in *H. arctochelys*

H. tetraneton:

All trees:

Fusion of xiphiplastra (9): present --> absent

Nuchal notch (67): anterior rim of nuchal convex or slightly notched --> anterior rim of carapace with deep nuchal notch, as developed in *H. rememdium*

Some trees:

Number of plastral callosities (7): seven, or five --> four

H. sterea:

All trees:

Width-length of nuchal bone (0): greater than 4 --> greater than 3

Point of reversal of orientation of neurals (14): at neural seven --> at neural four, five, or six

Perochelys lamadongensis:

Some trees:

Size of eighth pleurals (6): large --> reduced or absent

Pleurals which meet at midline (13): eighth only --> none

Suprascapular fontanelles (15): closed at hatching --> closed in large adults only, or open throughout life

Perochelys hengshanensis:

All trees:

No autapomorphies:

Adocus lineolatus:

All trees:

No autapomorphies:

Gobiapalone orlovi:

All trees:

Width-length of nuchal bone (0): greater than 3 --> greater than 4

Number of plastral callosities (7): four --> five

Postorbital bar relative to orbit (50): about equal to orbit to 1/3 of orbit --> less than 1/5 of orbit

Some trees:

Number of neurals (fused 1 and 2 counted as two) (11): eight --> eight or nine

Length epiplastra anterior to entoplastron contact (17): short --> intermediate

Quadratojugal participates in processus trochlearis oticum (51): yes --> no

Petrochelys kyrgyzensis:

Some trees:

Suprascapular fontanelles (15): closed in large adults only --> closed at hatching

Kuhnemys maortuensis:

Some trees:

Largest adult size 200 mm or less (disc length) (20): yes --> no

Node 35:

All trees:

No synapomorphies

Node 36:

All trees:

Anterior and posterior costiform processes of nuchal bone united (1): yes --> no

Epiplastron shape (16): J-shaped --> I-shaped

Carapace margin straight to concave posteriolaterally (21): no --> yes

Size of foramen palatinum posterius (32): small and divided --> many small openings

Strong dorsal processes on cervicals (54): no --> yes

Ischia extend into thyroid fenestra (62): no --> yes

Node 37:

All trees:

Size of foramen palatinum posterius (32): small --> small and divided

Epipterygoid contacts prootic posterior to foramen nervi trigemini (47): no --> yes

Coracoid longest of three pectoral processes (65): yes --> no

Hyoplastron with strongly serrated medial edge (84): absent --> present

Node 38:

All trees:

Hyoplastra and hypoplastra fuse just after hatching (8): no --> yes

Hypo-xiphiplastral union (10): xiphiplastra lateral to hypoplastra --> hypoplastra lateral to xiphiplastra

Epipterygoid fuses to pterygoid (48): never --> in subadults

Metischial processes present and distinct (63): yes --> no

Some trees:

Number of plastral callosities (7): five --> seven

Node 39:

All trees:

Total number of peripherals (4): 20 --> 14-18, or 0

Vomer divides maxillae (29): yes --> no

Foramen posterius canalis carotici interni relative to lateral crest of basioccipital tubercle (36): above --> below

Exoccipital contacts pterygoid (38): yes --> no

Mid-line contact of hyoplastra, hypoplastra and xiphiplastra (72): hyo-, hypo and xiphiplastra contact another fully along the entire mid-line in adults, as developed

in *H. rememdium* and *H. arctochelys* --> hyo-, hypo- and xiphiplastra do not contact another fully, even in adults

Proportions of costals VIII (82): significantly taller than wide --> wider than long to nearly square

Ilium with expanded dorsal end (83): present --> absent

Ossification of basibranchials (87): poorly ossified, especially posterior pair --> well ossified

Node 40:

All trees:

Pleurals which meet at midline (13): seventh and eighth or eighth only --> eighth only

Dorsal edge of aperture narium externum laterally emarginated (26): strongly --> no

Vomer divides maxillae (29): no --> yes

Foramen posterius canalis carotici interni relative to lateral crest of basioccipital tubercle (36): below --> in it

Epipterygoid, if present, contacts the palatine (43): yes --> no

Ventral keel on 8th cervical present and limited to posterior end (53): no --> yes

Strong dorsal processes on cervicals (54): no --> yes

Number of ossifications in corpus hyoidis (55): 8 --> 3

Ossifications of comu branchiale II broad and strongly sutured (57): no --> yes

Some trees:

Number of neurals (fused 1 and 2 counted as two) (11): eight --> eight or nine

Jugal contacts parietal on skull surface (24): no --> yes

Node 41:

All trees:

Point of reversal of orientation of neurals (14): at neural six --> at neural six or seven

Process on medial edge of hypoplastron (86): with enlarged anterior process separated by a gap from smaller posterior processes --> of subequal size and radiating outward from the medial edge of the bone

Node 42:

All trees:

First and second neurals fused (3): no --> yes

Suprascapular fontanelles (15): closed at hatching --> closed in large adults only

Foramen palatinum posterius forms in (33): palatine only --> palatine and pterygoid and/or maxilla

Foramen jugulare posterius (34): enclosed --> open

Mobility of entoplastron and anterior development of hyoplastron (74): lateral branches of entoplastron abut loosely against hyoplastron, but hyoplastron develops an anterior flap/shoulder, as seen in *Plastomenus* aff. *thomasii* --> lateral

branches of entoplastron abut loosely against hyoplastron, anterior rim of hyoplastron develops no anterior flap/shoulder

Hyoplastron with strongly serrated medial edge (84): absent --> present

Node 43:

All trees:

Width-length of nuchal bone (0): greater than 2 --> greater than 3

Number of plastral callosities (7): five --> four

Dorsal edge of aperture narium externum laterally emarginated (26): no --> strongly

Some trees:

Number of neurals (fused 1 and 2 counted as two) (11): seven or eight --> eight

Node 44:

All trees:

Number of neurals (fused 1 and 2 counted as two) (11): eight or nine --> nine

Some trees:

Pleurals which meet at midline (13): seventh and eighth or eighth only --> eighth only

Symphyseal ridge strong and present in a depression (59): no --> yes

Node 45:

All trees:

Length epiplastra anterior to entoplastron contact (17): short, or intermediate --> long

Largest adult size 200 mm or less (disc length) (20): no --> yes

Enclosed foramen jugulare posterius (35): enclosed by pterygoid --> enclosed by opisthotic

Basisphenoid shape (39): not medially constricted --> medially constricted

Some trees:

Number of neurals (fused 1 and 2 counted as two) (11): eight --> eight or nine

Node 46:

All trees:

Size of eighth pleurals (6): large --> reduced or absent

Vomer divides maxillae (29): no --> yes

Average ratio of intermaxillary foramen length to length primary palate (49): 0.20 to 0.40 --> about 0.60

Some trees:

Number of ossifications in comu branchiale II (56): 2-6 --> 7 or more

Node 47:

All trees:

Variability in position of neural reversal (12): always at same neural --> highly variable

Sexual dimorphism in disc length (22): no --> yes

Number of lateral hyoplastral processes (76): two --> one

Node 48:

All trees:

First and second neurals fused (3): yes --> no

Variability in position of neural reversal (12): always at same neural --> always at adjacent neurals

Point of reversal of orientation of neurals (14): at neural seven --> at neural six or seven

Some trees:

Pleurals which meet at midline (13): eighth only --> seventh and eighth or eighth only

Node 49:

All trees:

Number of plastral callosities (7): four --> seven

Suprascapular fontanelles (15): closed in large adults only --> open throughout life

Largest adult size 200 mm or less (disc length) (20): no --> yes

Contact between pterygoid and foramen nervi trigemini occurs when epipterygoid is present (44): yes --> no

Postorbital bar relative to orbit (50): about equal to orbit to 1/3 of orbit --> less than 1/5 of orbit

Angle of acromion process to scapula approaches that of coracoid to acromion (64): no --> yes

Node 50:

All trees:

Width-length of nuchal bone (0): greater than 3 --> greater than 4

Shape of deep portion of entoplastron (73): lateral branches of entoplastron more or less straight and merge anterior at a clear angle --> entoplastron wide and rounded, as seen in *P*. aff. *thomasii*

Node 51:

All trees:

Fusion of xiphiplastra (9): absent --> present

Dentary symphysis (80): short --> extremely long, mandible extremely elongate, as developed in *Plastomenus thomasii*

Some trees:

Point of reversal of orientation of neurals (14): at neural six --> no reversal

Node 52:

All trees:

Hypo-xiphiplastral union (10): xiphiplastra lateral to hypoplastra --> hypoplastra lateral to xiphiplastra

Depressions on eighth pleurals for contact of ilia (18): absent --> present

Bridge length (19): short --> long

Mid-line contact of hyoplastra, hypoplastra and xiphiplastra (72): hyo-, hypo- and xiphiplastra do not contact another fully, even in adults --> hyo-, hypo and xiphiplastra contact another fully along the entire mid-line in adults, as developed in *H. rememdium* and *H. arctochelys*

Proportions of costals VIII (82): wider than long to nearly square --> significantly taller than wide

Some trees:

Number of plastral callosities (7): four --> five

Node 53:

All trees:

Shape of neural 1 and 2 (neural 2 and 3 of Meylan 1987) (68): neurals 1 and 2 hexagonal with short posterior sides --> neural 1 circular to rectangular and neural 2 octagonal, as developed in *H. rememdium* and *H. arctochelys*

Node 54:

All trees:

Development of surface sculpturing of carapace and plastron (66): all metaplastic portions of carapace and plastron have trionychid sculpturing --> trionychid pattern grades towards the center of carapacial and plastral disk to a smooth pattern, as developed in *Hutchemys rememdium* and *Hutchemys arctochelys*

Splitting of costals along distal margin (69): costal rim rounded or graded --> dorsal rims split into separately protruding dorsal and visceral portions, as developed in *H. rememdium* and *H. arctochelys*

Mobility of entoplastron and anterior development of hyoplastron (74): lateral branches of entoplastron abut loosely against hyoplastron, but hyoplastron develops an anterior flap/shoulder, as seen in *Plastomenus* aff. *thomasii* --> entoplastron tightly integrated into anterior plastral lobe due to strong development of anterior flap/shoulder, as developed in *H. rememdium* and *H. arctochelys*

Some trees:

Point of reversal of orientation of neurals (14): no reversal --> at neural seven

Node 55:

All trees:

Development of surface sculpturing of carapace and plastron (66): all metaplastic portions of carapace and plastron have trionychid sculpturing --> plastron sculpture greatly subdued or absent

Some trees:

Largest adult size 200 mm or less (disc length) (20): no --> yes

Node 56:

Some trees:

Point of reversal of orientation of neurals (14): at neural seven --> at neural six

Serrated medial edge of hyoplastron extends nearly to the posterior edge of bone (85): absent --> present

Process on medial edge of hypoplastron (86): with enlarged anterior process separated by a gap from smaller posterior processes --> of subequal size and radiating outward from the medial edge of the bone

Supplementary References

Danilov, I. G., *et al*. Cretaceous soft-shelled turtles (Trionychidae) of Mongolia: new diversity, records and a revision. *J. Syst. Palaeontol*. **12**, 799–832 (2014).

Delfino, M., Scheyer, T. M., Fritz, U., & Sánchez-Villagra, M. R. An integrative approach to examining a homology question: shell structures in soft-shell turtles. *Biol. J. Linn. Soc.* **99**, 462–476 (2010).

Hutchison, J. H. New soft-shelled turtles (Plastomeninae, Trionychidae, Testudines) from the Late Cretaceous and Paleocene of North America. *Paleobios* **29**, 36–47 (2009).

Joyce, W. G., Lyson, T. R., & Williams, S. New cranial material of *Gilmoremys lancensis* (Testudines, Trionychidae) from the Hell Creek Formation of southeastern Montana, USA. *J. Vert. Paleontol.* **36**, e1225748 (2016).

Joyce, W. G., Revan, A., Lyson, T. R. & Danilov, I. G. Two new plastomenine softshell turtles from the Paleocene of Montana and Wyoming. *Bull. Peabody Mus. Nat. Hist.* **50**, 307–325 (2009).

Joyce, W. G. & Lyson, T. R. New material of *Gilmoremys lancensis* nov. comb.(Testudines: Trionychidae) from the Hell Creek Formation and the diagnosis of plastomenid turtles. . *J. Paleontol.* **85**, 442–459 (2011).

Li, L., Joyce, W. G. & Liu, J. The first soft-shelled turtle from the Jehol Biota of China. *J. Vert. Paleontol.***35**, e909450, DOI: 10.1080/02724634.2014.909450 (2015).

Liebing, N., *et al*. Molecular phylogeny of the softshell turtle genus *Nilssonia* revisited, with first records of *N. formosa* for China and wild-living *N. nigricans* for Bangladesh. *Vert. Zool*. **62**, 261–272 (2012).

Li, H.*et al*. Phylogenetic relationships and divergence dates of softshell turtles (Testudines: Trionychidae) inferred from complete mitochondrial genomes. *J. Evol. Biol.* DOI: 10.1111/jeb.13070 (2017).

Le, M., *et al*. A phylogeny of softshell turtles (Testudines: Trionychidae) with reference to the taxonomic status of the critically endangered, giant softshell turtle, *Rafetus swinhoei*. *Org. Divers. Evol.* **14**, 279–293 (2014).

Meylan, P. A. The phylogenetic relationships of soft-shelled turtles (family Trionychidae). *Bull. Am. Mus. Nat. Hist.* **186**, 1–101 (1987).

Nessov, L. A. On some Mesozoic turtles of the Fergana depression (Kyrgyzstan) and Dzhungar Alatau ridge (Kazakhstan). *Russ. J. Herpetol.* **2**, 134–141 (1995).

Sheil, C. A. Osteology and skeletal development of *Apalone spinifera* (Reptilia: Testudines: Trionychidae). *J. Morphol.* **256**, 42–78 (2003).

Siebenrock, F. Ueber den Bau und die Entwicklung des Zungenbein-Apparates der Schildkröten. *Ann. Naturhist. Mus. Wien* **13**, 424–437 (1898).

Yeh, H.-K. New materials of fossil turtles of Inner Mongolia. *Vert. PalAs*. **9**, 47–78 (1965).
